# A hypothesis of sudden body fluid vaporization in the 79 AD victims of Vesuvius

**DOI:** 10.1371/journal.pone.0203210

**Published:** 2018-09-26

**Authors:** Pierpaolo Petrone, Piero Pucci, Alessandro Vergara, Angela Amoresano, Leila Birolo, Francesca Pane, Francesco Sirano, Massimo Niola, Claudio Buccelli, Vincenzo Graziano

**Affiliations:** 1 Laboratory of Human Osteobiology and Forensic Anthropology, Department of Advanced Biomedical Sciences, Azienda Ospedaliera Universitaria “Federico II” 5, Naples, Italy; 2 Department of Chemical Sciences, University of Naples “Federico II”, Complesso Monte Sant'Angelo, Naples, Italy; 3 CEINGE Biotecnologie Avanzate S.C a R.L., Naples, Italy; 4 Task Force di Ateneo "Metodologie Analitiche per la Salvaguardia dei Beni Culturali", Universita' di Napoli Federico II, Naples, Italy; 5 Parco Archeologico di Ercolano, Corso Resina Herculaneum, Naples, Italy; 6 Department of Advanced Biomedical Sciences, Azienda Ospedaliera Universitaria “Federico II”, Naples, Italy; University of Otago, NEW ZEALAND

## Abstract

In AD 79 the town of Herculaneum was suddenly hit and overwhelmed by volcanic ash-avalanches that killed all its remaining residents, as also occurred in Pompeii and other settlements as far as 20 kilometers from Vesuvius. New investigations on the victims' skeletons unearthed from the ash deposit filling 12 waterfront chambers have now revealed widespread preservation of atypical red and black mineral residues encrusting the bones, which also impregnate the ash filling the intracranial cavity and the ash-bed encasing the skeletons. Here we show the unique detection of large amounts of iron and iron oxides from such residues, as revealed by inductively coupled plasma mass spectrometry and Raman microspectroscopy, thought to be the final products of heme iron upon thermal decomposition. The extraordinarily rare preservation of significant putative evidence of hemoprotein thermal degradation from the eruption victims strongly suggests the rapid vaporization of body fluids and soft tissues of people at death due to exposure to extreme heat.

## Introduction

Vesuvius is an active volcano situated about 12 kilometers from Naples, one of the metropolitan cities at highest risk in the world, with its population of more than three million [1]. Archaeological and volcanological site evidence show that Vesuvius tends to have a major (Plinian) eruption at least every 2,000 years [2–4]. In AD 79 a sudden Plinian event with subsequent volcanic pumice fallout and ash-avalanches affected an extensive area, causing total devastation and thousands of victims [5]. The initial fallout phase (pumice air-fall phase), driven by the dominant southerly and south easterly winds [6], was dispersed up to a distance of about 70 kilometers [7]. The later pyroclastic surges and flows (rapid gravity-driven currents of volcanic ash and hot gases generated by the collapse of the Plinian eruptive column) reached up to 30 kilometers northwest and west of Vesuvius [1,6,8].

In the early phase of the eruption the first fatalities occurred in Pompeii as a result of roofs and floors collapsing due to pumice accumulation [9,10]. In the next hours, the remaining inhabitants of Herculaneum (ca. 4–5,000) [11,12] and Pompeii (ca. 20,000) [13,14], as well as those from nearby settlements and villas (e.g. Villa B at Oplontis) [15–17], who were not able to evacuate in time, were overwhelmed by the hot surge clouds [18,19].

At Herculaneum, 300 people who had taken refuge in 12 waterfront chambers along the beach (S1 Fig) were suddenly engulfed by the abrupt collapse of the rapidly advancing first pyroclastic surge (S1) [8] (S2 Fig). In just a few hours the towns of Herculaneum, Pompeii and Stabiae, situated respectively about 7, 10 and 16 kilometers from the vent, were definitively buried by subsequent pyroclastic currents [6], whose eruptive deposits reached a maximum thickness of 20 meters [8,20–22]. In this area, the archaeological investigations of the last three centuries brought to light several Roman settlements and hundreds of human victims, even at a distance of 20 kilometers as far as Stabiae and close suburban villas in Gragnano [19,21,22].

## Background

### Archaeological and osteological context

New excavations started in the 80s after the casual discovery of human remains in the suburban area of Herculaneum. On the beach and in 6 of the 12 waterfront chambers were uncovered approximately 140 victims of the eruption [23]. These skeletons, initially recovered and studied by Sara Bisel [24–26], were later the subject of several bioanthropological studies [27–35]. Further archaeological investigations conducted in the early 90s in 6 of the chambers not yet excavated brought to light an additional large group of human victims, left untouched within the ash surge deposit [36]. In the second half of the 90s, these skeletal remains were the subject of a valorization project through the making of fiberglass casts [37,38]. The latter were later placed in the original context of discovery, after archaeological investigation and final recovery of the victims’ skeletons leaded by one of the authors (P. Petrone) [38,39]. The joint bioarchaeological and taphonomic study of the skeletons prior their removal allowed the investigators to verify the interactions between victims’ bodies and the volcanic ash deposit. The resulting new site evidence combined with those from laboratory bones analysis allowed to obtain new information on the causes of death and the heat-induced effects on people [40–43]. Further bioanthropological and paleopathological studies were also carried out on the same skeletal sample [44–47].

### Volcanological context

Recent global eruptions show that pyroclastic density currents are the greatest threat to life [48–52] and thus the dominant hazard in densely populated areas [53]. A dilute pyroclastic density current or surge is typically an intensely hot (200–500 °C) fast-moving cloud (100 to 300 km/hr) of fine ash, in an environment being low in free oxygen content and rich of superheated steam and other volcanic gases [6,48]. In such conditions survival is likely to be impossible, particularly due to the intense heat reached in areas closer to the vent [54]. In pyroclastic density currents, thermal injury may be at least as important as asphyxia in causing immediate death [48].

In the main body of a proximal surge temperatures may be as high as 400–500 °C, whereas in distal regions temperatures of 200–300 °C are more common [48,54]. These temperatures are analogous to those of the 79 AD pyroclastic surges that hit first Herculaneum and later Pompeii, as determined with various methods, including TRM on lithic clasts [55–58], bone analysis vs heating experiments [42], and charcoal reflectance [59].

Pyroclastic surge clouds are responsible for emplacement of the largest and widespread ash deposit in the suburban area of Herculaneum [6,8]. Rapid deposition of extremely fine-grained ash into thermally stratified volcanic deposit [60] on the beach and within the boat-chambers, and abrupt entrapment and burial of the victims by the hot ash surge [6,8] could have protected them from being bioturbated [61], thus resulting in the exceptional preservation of fully articulated skeletons in the last vital posture [5,39,40,48].

### Heat effects and causes of death

The effects of the eruption on the inhabitants of Herculaneum and Pompeii as well as on the people living in the other urban and suburban settlements around the volcano have been the subject of several studies [5,9,14,19,21,27,28,39–42,48]. Apart from the casualties occurred in the initial pumices fallout phase by buildings collapse in Pompeii [10,19,21], studies on the causes of death are mostly referred to the effects of heat associated with the pyroclastic surge clouds emplacement in both Herculaneum [41–43,49] and Pompeii [14,48].

Although the heat of the ash surges has been mostly accepted as a major cause of mass mortality in the 79 AD eruption, there are some differences in interpretation depending on the distance from the volcano and, within the same site, on the place where victims were found. As regards Herculaneum, more recent studies agree on the rapid death of people discovered on the sea shore area [34,39–43], but some authors hypothesized a gradient of heat-induced effects. So, even if nearly every skeleton had some evidence of bone thermal exposure (changes in color, charring, fracturing) [28,34,40,42], the few victims found on the beach were assumed to show greater thermal effects compared to those sheltered inside the chambers [27,34]. Based on the previous assumption, it was also hypothesized that death was instantaneous only for people found on the beach, while those refugees in the chambers would have died from asphyxiation [27,28]. In fact, a comparative analysis of the full skeletal sample has not yet been achieved, being the victims’ samples from different excavation surveys (conducted in the 80s by S. Bisel, and in 1997–1999 by P. Petrone) [26,39] studied separately. With regard to previous interpretations on the causes of death, also at a greater distance as in Pompeii, death by asphyxiation in both pumices fallout and pyroclastic surge phases has long remained the most accredited hypothesis [9,13,14,21,62].

**Fig 1:**
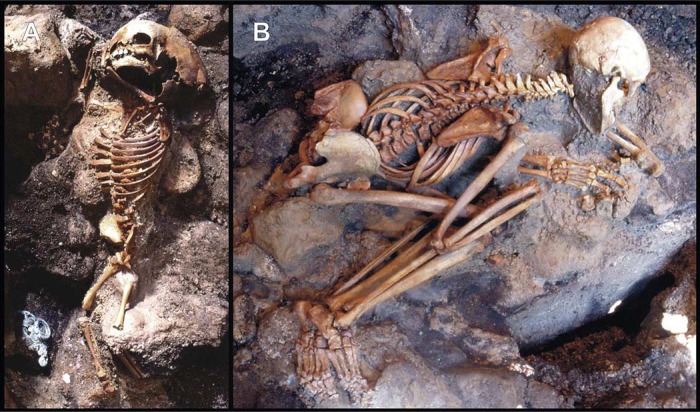
Human victims discovered on the sea-shore area. Skeletons showing "life-like" stance: a child (A) (Ind. 41) and young adult male (B) (Ind. 22) unearthed from the ash surge deposit (chamber 10) (S1 Table). The child’s corpse displays flexure only of the upper limbs, indicative of an incipient “pugilistic attitude”. Full exhibit of this heat-induced stance is never found in the victims' corpses discovered at Herculaneum.

The latter accounts are at variance with a first forensic interpretation concerning the victims found in the surge deposit [48], as well as with more recent multidisciplinary studies. Taphonomic, bioanthropological and volcanological site investigations and laboratory evidence [40,42], coupled with results from heating experiments on recent human bone samples [41], have shown that the Herculaneum residents were instantly killed by the extreme high temperature of the emplacing S1 surge, although it was previously believed that death had occurred by slow suffocation from ash inhalation. Skull and bone charring and cracking, as well as instant hand and foot contraction (flexor reflex by the nociceptive C fibers) [63] and spine hyperextension, have been described as thermally induced major effects on the victims' skeletons unearthed from both the beach and the sea-front chambers [27,28,34,40,42,43]. Histological and ultra-structural investigation have revealed linear and polygonal cracking of the intra- and inter-osteonic structure associated with incipient recrystallization (S3 Fig), bone changes typically induced by heat [64,65]. Evidence of sudden death is provided by the victims' corpses, appearing to be "frozen" in the last vital action (life-like stance) (Fig 1). The lack of voluntary self-protective reaction or agony indicates that any vital activity had to stop within a time shorter than the conscious reaction time, a state known as fulminant shock [66]. The widespread occurrence of life-like stance has been found consistent with cadaveric spasm, a rare but diagnostic form of instantaneous muscular stiffening (instant *rigor*), induced by instant thermal coagulation in victims from pyroclastic currents [48], which crystallizes the last vital activity prior to death [67,68]. As also observed in the other sites buried by the 79 AD eruption, the overall evidence including the predominance of life-like stance suggests the occurrence of thermally-induced instant death of the inhabitants in the Vesuvius area up to at least 20 kilometers from the vent [5,40–43,48].

A thorough investigation on the Herculaneum victims' skeletons and their ash (hereafter, the term “ash” is always referred to volcanic ash) burial context revealed the preservation of atypical mineral residues encrusting the bones, which also impregnate the ash filling the skulls and the ash-bed. The iron and iron oxides amounts detected by inductively coupled plasma mass spectrometry and Raman microspectroscopy suggest such residues to be the final products of heme iron upon thermal decomposition. The significant putative evidence of hemoprotein thermal degradation and additional evidence of heat-induced effects seem to suggest the rapid vaporization of body fluids and soft tissues of victims resulting from exposure to the extreme high temperature of the ash-avalanches.

## Materials and methods

The archaeological site of Herculaneum is placed at the foot of Vesuvius, at about six kilometers from metropolitan Naples. In 1997–1999, a large group of about 80 skeletons of victims of the 79 AD Vesuvius eruption were recovered from the ash surge deposit inside chambers 5, 10, 11, and 12 on the seafront area of the town [37–42]. Two subsequent archaeological excavation campaigns were conducted by one of the authors (P. Petrone), in collaboration with the director of the Herculaneum archaeological site (M. Pagano, Superintendence of Pompeii). The skeletal remains were carefully examined in the laboratory for the presence of certain mineral residues, observed for the first time during the archaeological survey, which impregnated the ash burial deposit or encrusted the bones. Bones and ash samples were observed with a 10x-30x magnifying glass. Particular attention was also given to the detection of mineral residues from the ash filling the skulls. In order to avoid potential taphonomic complications, such residues were sampled only from those encrustations that affected the bones and the ash layers not at direct contact with metal artifacts like coins, rings and other types of personal objects found close to the victims [69]. As regards demographics and place of discovery of each skeleton, sex, age at death and chamber numbering are reported in S1 Table (see also [41]). Type and source of each of the 103 samples detected for iron content (Table 1) are specified in S2 Table. All necessary permits were obtained for the study of the Herculaneum specimen, which was approved by the Ethics Committee for Biomedical Activities of the Azienda Ospedaliera Universitaria (AOU) Federico II (Protocol 101/17).

**Table 1:** ICP-MS results. Data collected from 103 samples, integrated by using a proper calibration curve.

Sample	Fe (mg/kg)	Sample	Fe (mg/kg)	Sample	Fe (mg/kg)
C1	3240.9	C36	338.2	C71	22149.6
C2	1043.4	C37	696.2	C72	61234.4
C3	3320.5	C38	707.0	C73	19456.3
C4	3301.6	C39	8257.3	C74	67124.0
C5	81037.8	C40	21890.6	C75	86945.4
C6	2521.3	C41	972.1	C76	64188.1
C7	5266.7	C42	872.1	C77	75114.3
C8	1709.2	C43	1618.5	C78	59428.3
C9	1834.7	C44	31859.2	C79	73743.9
C10	918.4	C45	32418.5	C80	77698.4
C11	1485.1	C46	30895.4	C81	62474.1
C12	1144.2	C47	1739.5	C82	69846.4
C13	654.0	C48	1356.6	C83	75633.0
C14	2804.1	C49	71563.1	C84	66408.3
C15	3083.4	C50	58519.0	C85	41369.0
C16	6521.2	C51	411.7	C86	30489.3
C17	832.2	C52	6258.5	C87	70896.5
C18	496.3	C53	6974.4	C88	50206.0
C19	2322.2	C54	7015.7	C89	846.0
C20	12892.0	C55	6549.3	C90	52477.1
C21	302.0	C56	18695.6	C91	542.7
C22	75593.1	C57	6874.2	C92	737.0
C23	355.3	C58	6974.1	C93	62881.7
C24	489.6	C59	1456.0	C94	69743.6
C25	44370.0	C60	204.3	C95	67452.3
C26	34896.3	C61	996.4	C96	48796.4
C27	55586.7	C62	1281.7	C97	75632.5
C28	1001.2	C63	375.1	C98	71633.0
C29	1127.0	C64	1283.8	C99	76455.3
C30	16719.8	C65	746.7	C100	62489.4
C31	7745.6	C66	755.0	C101	74528.4
C32	829.9	C67	1898.8	C102	68049.2
C33	9089.6	C68	1367.5	C103	8125.7
C34	15846.4	C69	51296.4		
C35	726.7	C70	47633.0		

### Inductively coupled plasma mass spectrometry (ICP-MS)

A selection was made to investigate the presence of iron [70] from 103 different archaeological samples. The samples collected were differentiated by the presence of red or black residuals. Each sample (10 mg) was digested in acid in a Teflon vessel in a microwave oven (Milestone Ethos 900-Mega II). Digestion was obtained by adding a mixture of 2 mL of 67% HNO3 and 4 mL of 37% HCl. HNO3 and HCl were Super Purity Solvent grade from Romil, Cambridge, UK. Acidic mineralization was achieved with the following microwave oven program: 20 min to reach 220°C at 1400 W; 15 min at 220°C and 1400 W; ventilation for 30 min. The solution was then quantitatively transferred into polystyrene liners and stored at 4°C until ICP-MS analysis was performed. The analyses were carried out in triplicate on an Agilent 7700 ICP-MS, equipped with a frequency-matching RF generator and 3rd generation Octopole Reaction System (ORS), operating with helium as cell gas on diluted samples (1:10 v/v Milli-Q water). The parameters were set as follows: radiofrequency power 1550 W, plasma gas flow 14 L min-1; carrier gas flow 0.99 L min-1; He gas flow 4.3 mL min-1. The Octopole Reaction System was activated to improve metal quantification because of the interferences by polyatomic species produced by a combination of isotopes from plasma, reagents and matrix. Multi-element calibration standards were prepared in 5% HNO3 at four different concentrations (1, 10, 50, and 100 μg L−1). The standard addition approach for calibration on four concentration levels was used in order to keep the matrix-induced variations to a minimum. At least three replicates of each calibration standard were run. Moreover, in order to correct possible instrumental drifts, 103Rh was used as an internal standard (final concentration: 50 μg L−1). The error in the determination of the iron amount within the samples is within 10%.

### Raman microspectroscopy

Of the 103 samples, 22 were investigated by Raman microspectroscopy in order to detect, quantify and discriminate the possible preservation of heme and heme degradation products [71]. Raman analysis of these samples selected on the basis of iron content data analyzed by ICP-MS was performed to identify or exclude various species containing iron and identify other non-ferrous species. A confocal Raman microscope (Jasco, NRS-3100) was used to obtain Raman spectra. The 514 nm line of an air-cooled Ar + laser (Melles Griot, 35 LAP431 220) or a 647 nm line of a water-cooled Kr+ laser (Coherent) was injected into an integrated Olympus microscope and focused to a spot diameter of approximately 3 μm by a 20x objective with a final 4 mW power at the sample. A holographic notch filter was used to reject the excitation laser line. Raman backscattering was collected using a diffraction lattice of 1200 grooves/mm and 0.01–0.20 mm slits, corresponding to an average spectral resolution up to 1 cm^-1^. Typically, it took 60 s to collect a complete dataset from a Peltier-cooled 1024x128 pixel CCD photon detector (Andor DU401BVI). Raman measurements were finally triplicated for the purpose of reproducibility for each spot sampled. Wavelength calibration was performed by using cyclohexane as a standard.

### Proteomic analyses

Samples were treated in heterogeneous phase with different pre-treatments (either incubation with 6M urea, or extraction with TFA 0.1%, acetonitrile 10%, or extraction with RIPA buffer, or extraction with CH_3_CL_3_/CH_3_OH [6: 3; v/v]). This phase was followed by enzymatic digestion with trypsin at 37°C for 16 hours, purification using a reverse-phase C18 Zip Tip pipette tip (Millipore), and nano LC-MS/MS analysis on a CHIP MS 6520 QTOF equipped with a capillary 1200 HPLC system and a chip cube (Agilent Technologies, Palo Alto, CA) [72]. Raw data were used for protein identification with a licensed version of MASCOT software (www.matrixscience.com) version 2.4. with 10 ppm MS tolerance and 0.6 Da MS/MS tolerance; peptide charge from +2 to +3. No fixed chemical modification was inserted, but possible oxidation of methionines, deamidation at asparagines and glutamines, and the addition of hydroxylation on prolines and lysines were considered as variable modifications to query the SwissProt database, with *Homo sapiens* as a taxonomy restriction.

## Results

In the present work, in the wake of previous taphonomic, bio-anthropological and stratigraphic surveys within the seafront chambers on the beach of Herculaneum, the victims' skeletons and their ash burial context (S1 ash surge deposit) are examined in depth. The initial site investigation allowed to detect several features related to the sudden impact of the hot S1 ash surge on the people such as the life-like stance of bodies, the hyperflexion of the extremities, the spine hyperextension and the skeletons floating within the ash deposit. Moreover, the preliminary analysis of the bones highlighted some features typical of exposure to high temperature like color changes, carbonization and cracking.

**Fig 2:**
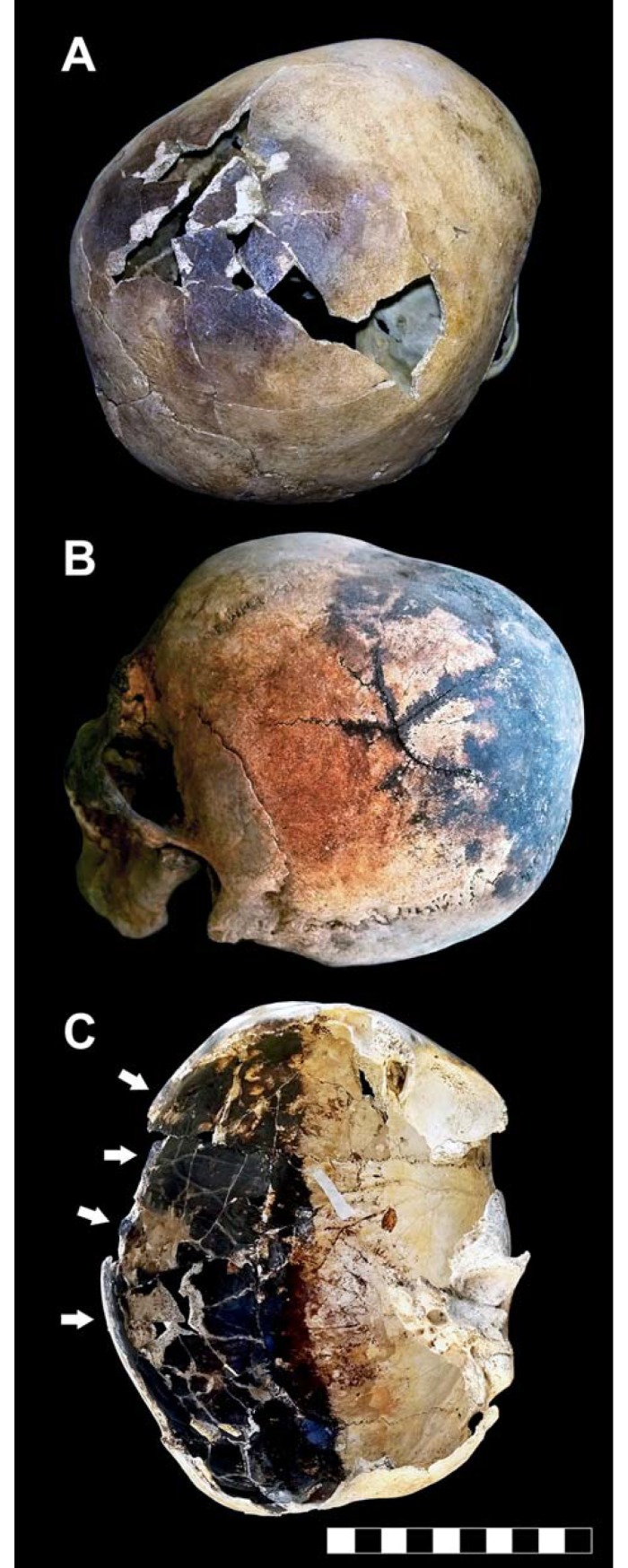
Thermal effects in human victims' skeletons. A. Skull of an older-aged adult male showing a dark stained and cracked parietal bone (ind. 11, chamber 12). B. Skullcap affected by a “stellate” fracture consisting of several cracks which radiate from a common center, characterized by charred outer margins (adult male, ind. 31, chamber 10). C. Exploded skull showing a partly dark stained inner table (right side, adult male, ind. 6, chamber 12); charring of the fractured margins is evident (white arrows) (bar scale 10 cm). The skull of this victim was lying in the ash bed on its left side.

**Fig 3:**
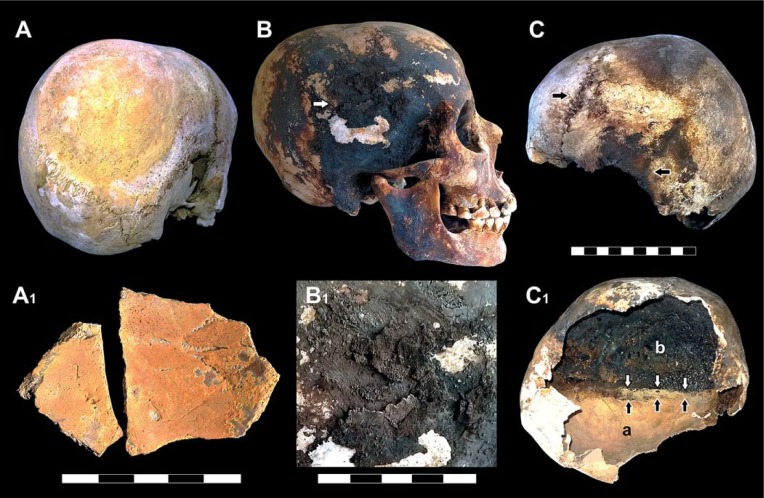
Red and black mineral incrustations detected in the victims' skulls. A. Child's skull showing a round area of thick red mineral residues encrusting the right parietal bone (ind. 18, chamber 12); A_1_. Inner bone surface of parietal fragments encrusted by red mineral residues (sporadic skull fragments, adult, chamber 10); B. Skull showing dark staining and black residues encrusting (white arrow) the parietal and temporal bones (B_1_) (adult male, ind. 31, chamber 10); C. Skullcap of a young individual displaying spotted dark stained areas and charred open sutures (black arrows) (ind. 29, chamber 12). The intracranial cavity (C_1_) shows a clear boundary (black and white arrows) between an inner table of unchanged color (a) next to a black stained one (b) (scale bars in cm).

Here we describe key evidence of body exposure to extreme heat provided by recurrent skull explosion (Fig 2), as detected by clear-cut fractures (Fig 2A), whose margins are sharp like those seen in cremated bones. In several cases, fracture lines radiate from a common center, thus showing a "stellate" appearance (Fig 2B). A single skull may also be affected by multiple fracture centers. Dark staining is typically associated with these cracked areas, whose exposed surface is charred as well (Fig 2C). Spotted areas of dark stains affecting the outer bone can also be found in intact skulls (Fig 3B and 3C). Fractures are always limited to the charred bone areas.

**Fig 4:**
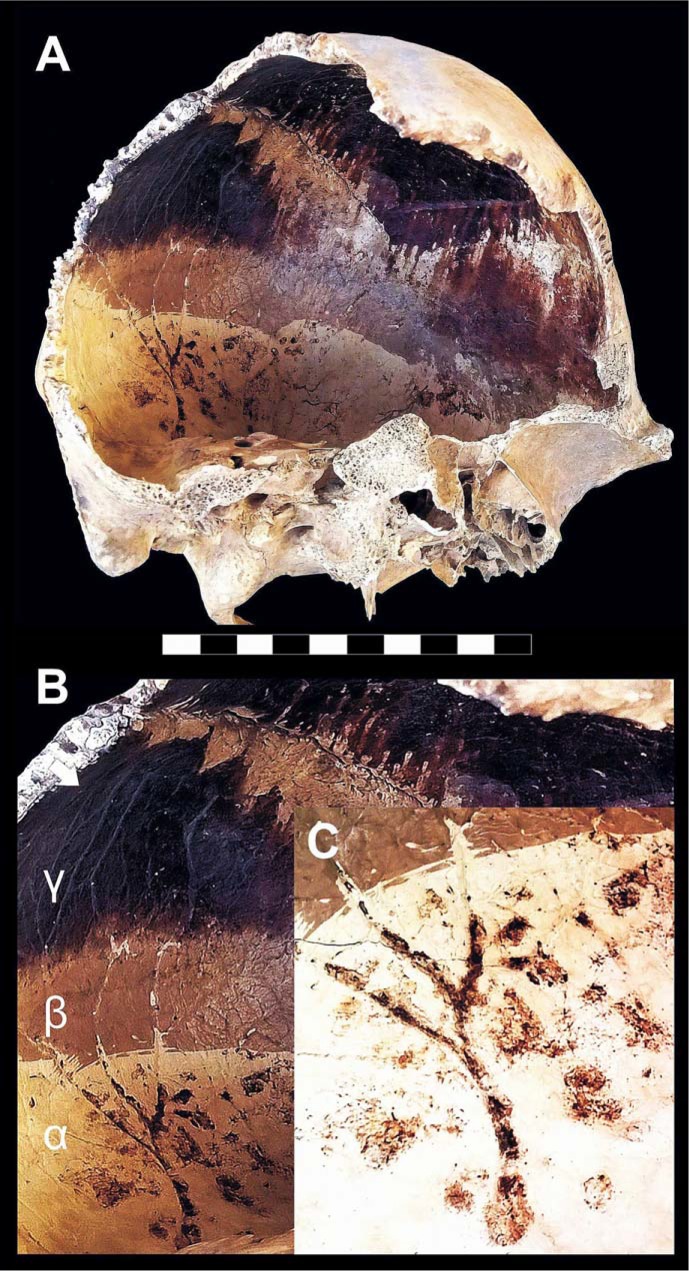
Thermal effects on the cranial cavity. A. Skull showing dark staining of the intracranial bone table (adult male, ind. 9, chamber 12). B. The inner bone surface progressively changing from pale yellow to black (α–γ). C. Brown residues encrusting the vascular grooves (scale bar in cm). Bone colors are based on the Munsell soil color chart [73].

A recurrent feature is the dark staining of the inner table beside a zone of unchanged color, with a distinct blackened/non-blackened pattern (Fig 2C), often associated with a typical effect of dark staining "exuding" from sutures. This feature is apparent close to the bone openings such as non-fused sutures (Fig 3C), orbits and the acoustic meatus (Fig 3B), the squamous part of the parietal bone (Fig 3B, panel 1 and 3C), the *foramen magnum*, and the margins of the skullcap fractures as well (Fig 2A, 2B and 2C). In some cases dark staining of the intracranial cavity appears to be gradual, the bone progressively appearing natural/pale yellow (α), bright brown (β), dark brown and black (γ) (Fig 4A and 4B) [73]. Pale yellow is indicative of minor thermal exposure (≤ 200 °C), whereas darker bone coloration matches with higher temperatures (300 to 400–500°C) [41,64,74–76].

The careful examination of the skulls first on the site and then in the laboratory showed that the presence of blackened bone near bone of unchanged color depends on the victim's posture at death: whatever the orientation of the head, the color of the downward-facing bone stays unchanged, whereas the upward-facing bone is mostly blackened (Fig 3C, panel 1). This evidence is concomitant with the pattern of bone cracking, since dark stained areas often exhibit fissure fractures, or may even be exploded, while those of unchanged color are never affected by such major heat effects. A substantial feature associated with the charring of the inner cranial table is the brown coloration of the vascular grooves (*sulci arteriosi* and *sulci venarum*) (Fig 4C). In general, as detected from the death assemblage, victims' skeletons from less crowded chambers show greater heat effects (i.e., bone charring and cracking, skull explosion) than those where people were crammed into the available space (S1C Fig).

**Fig 5:**
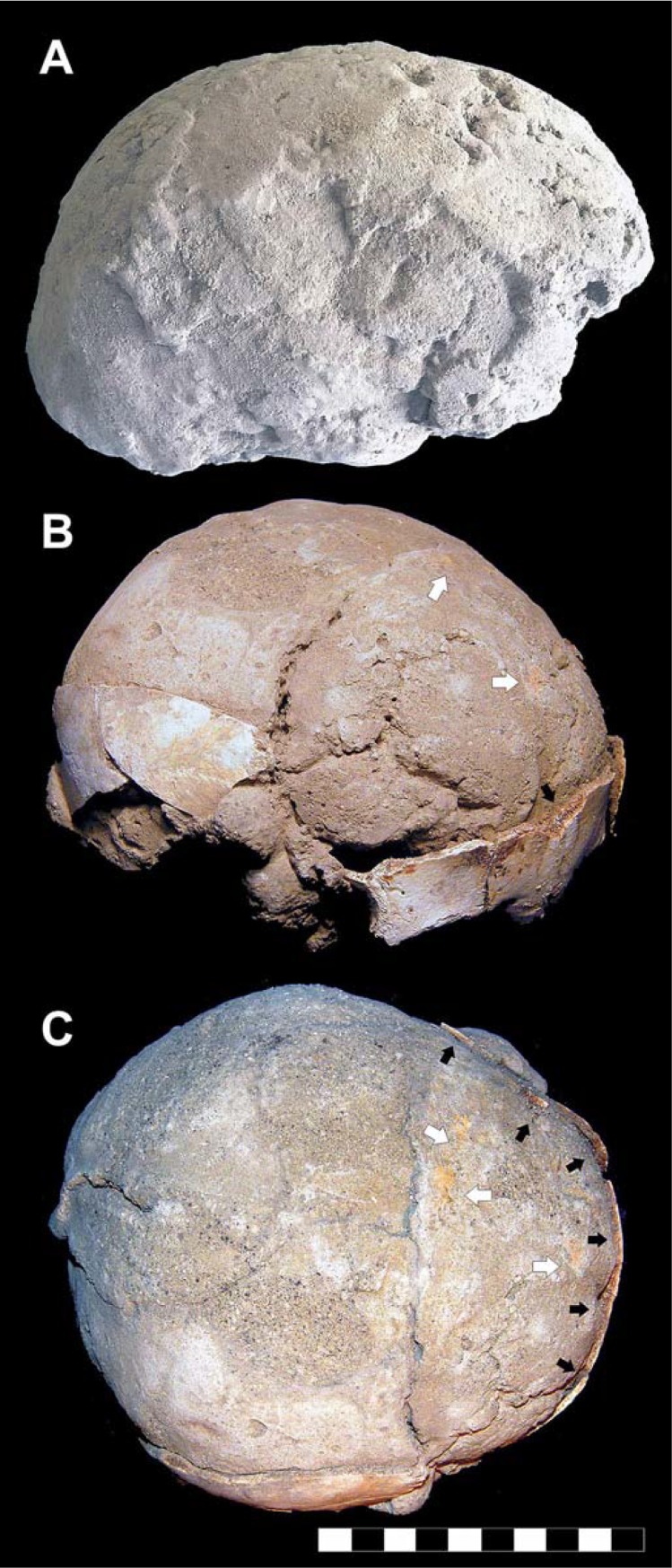
Brain ash casts from the victims' skulls. A. Brain-like ash cast from a skull of an adult male (ind. 4, chamber 12). B. Exploded skull of a child filled by ash, from one of the rare skeletons preserved from the early 1900s' excavations of the town (ind. A, weaver's house). C. Perfect replica of the brain shape is apparent from the imprint of the coronal and sagittal open sutures (C). As also detected in the victims discovered in the sea-front area, the ash is impregnated by red mineral residues (B, C, white arrows), which also encrust the exposed bone margins (B, C, black arrows). Some reddish-brown stained areas affect the outer bone surface (B, right supraorbital region) (scale bar in cm).

**Fig 6:**
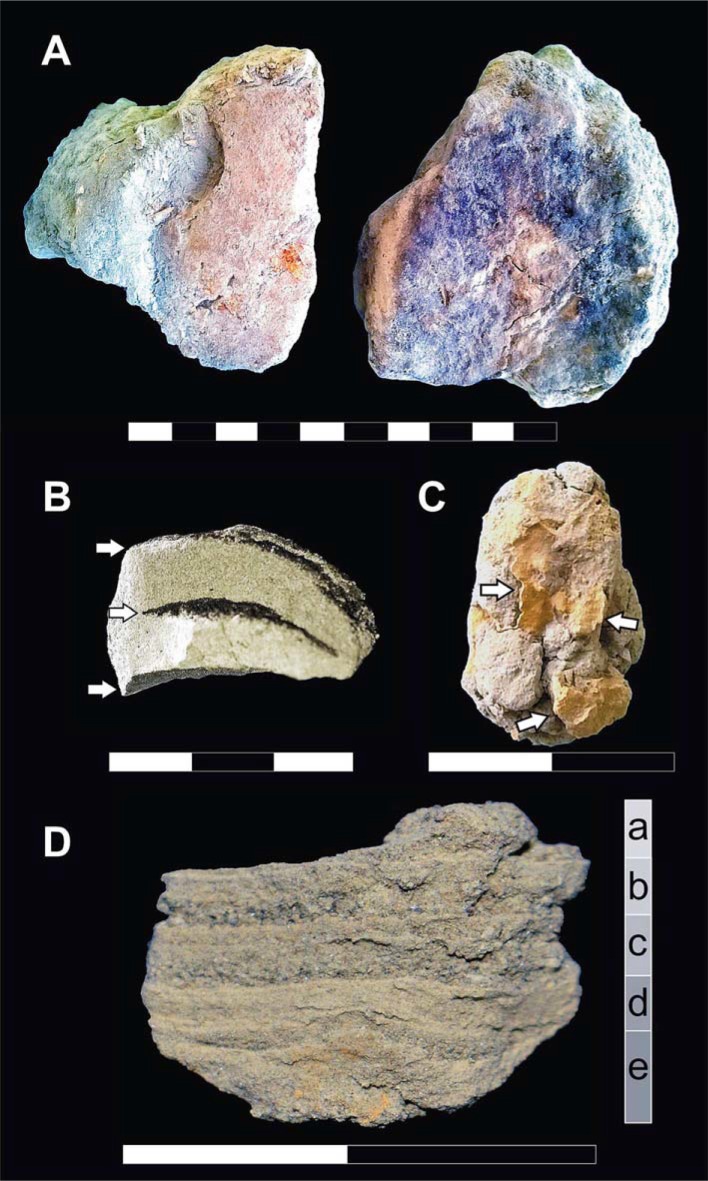
Micro stratigraphic evidence from the ash filling the skull cavity. A. Layers of red and dark red mineral residues impregnate the ash surface facing the unfilled intracranial space; B. Black residues impregnate ash by different layers; C. Red residues impregnating the ash mass mostly appear in the form of spotted inflorescences. D. Close view of an intracranial ash sample showing evident micro lamination by subsequent layers (a-e) of fine-grained ash. Note the red residues impregnating layer e (scale bars in cm).

An extraordinary find concerns skulls filled with ash, which indicates that after evaporation of the organic liquids the brain was replaced by ash (Fig 5). The presence of such an ash cast in all victims, even those showing minor heat effects, provides evidence that the S1 surge was sufficiently hot and fluid to penetrate the intracranial cavity soon after soft tissues and organic fluids disappeared, as supported by palaeomagnetic thermal measurements [56] and intracranial micro stratigraphy, the latter showing lamination by successive apposition of thin ash layers (Fig 6D).

**Fig 7:**
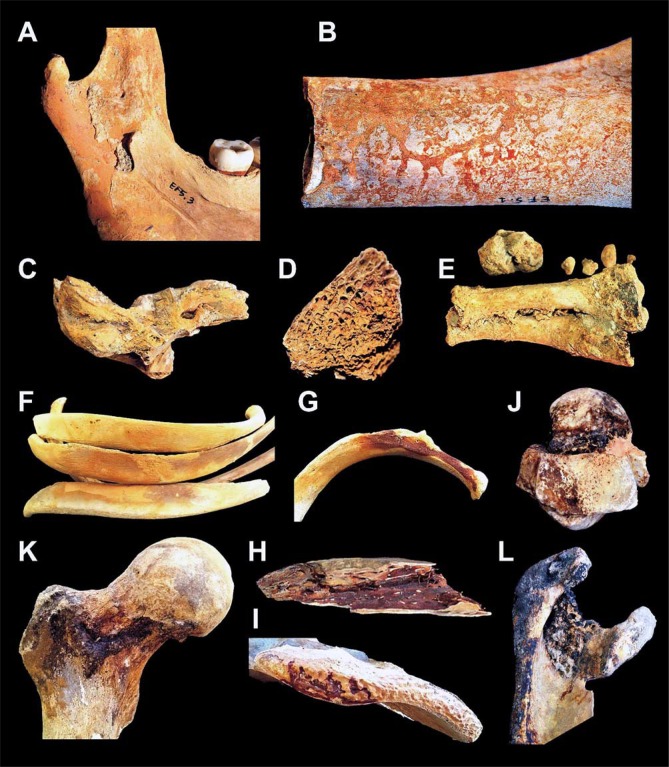
Red and black mineral residues detected on human victims' bones. Red incrustations: A. Jaws (adult, ind. 3, chamber 5); B. Femur diaphysis (young adult, ind. 3, chamber 5); C. Temporal and petrous bone (adult, sporadic bones, chamber 10); D. Proximal epiphysis of tibia (adult, sporadic bone, chamber 10); E. Metatarsal foot bones (adult, sporadic bones, chamber 10); F. Ribs (adult, ind. 1, chamber 12). Dark red incrustations: G, H. Ribs (adult, sporadic bones, chamber 10); I. Pubic symphysis (subadult, ind. 20, chamber 12). Black incrustations: J. Anklebone (adult, ind. 1, chamber 5); K. Femur (adult, ind. 1, chamber 5); L. Scapula (adult, ind. 11B, chamber 10).

**Fig 8:**
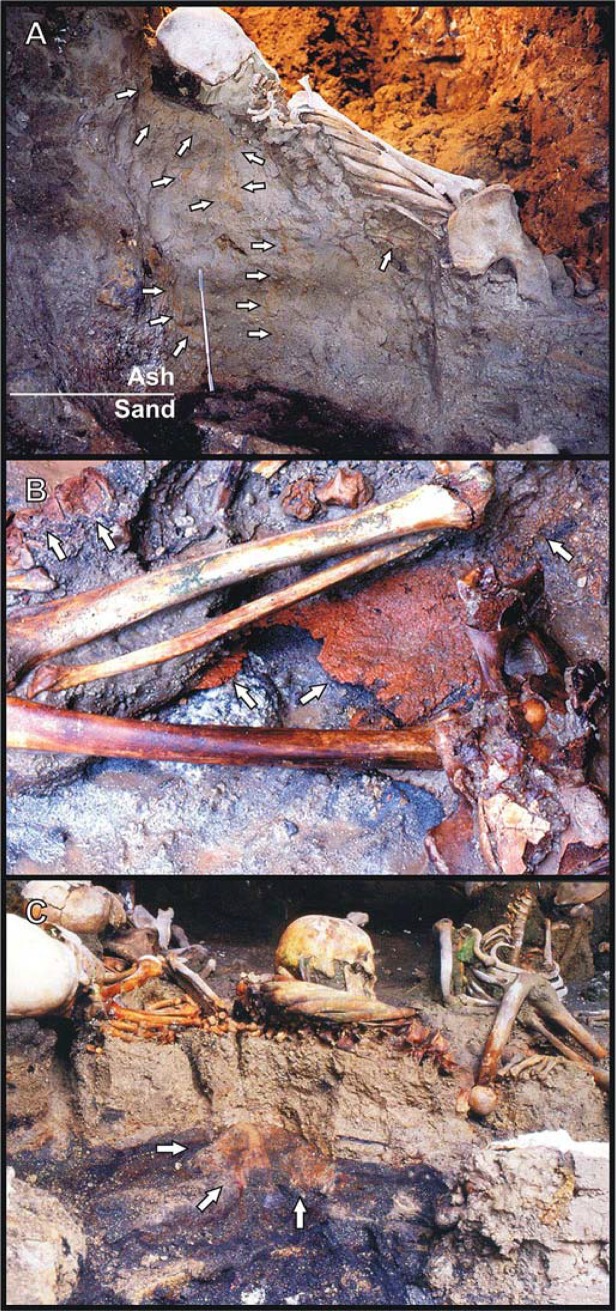
Evidence of red mineral residues detected on the archaeological site. Victims' skeletons in their original context during excavation (chambers 10, 12): A. Red mineral residues impregnating the ash bed deposit at different depths (white arrows); B. Thick layer of red mineral residues at the contact surface of the skeleton with the ash deposit (white arrows); C. Red residues may locally impregnate the sand, which appears mostly blackened.

**Fig 9:**
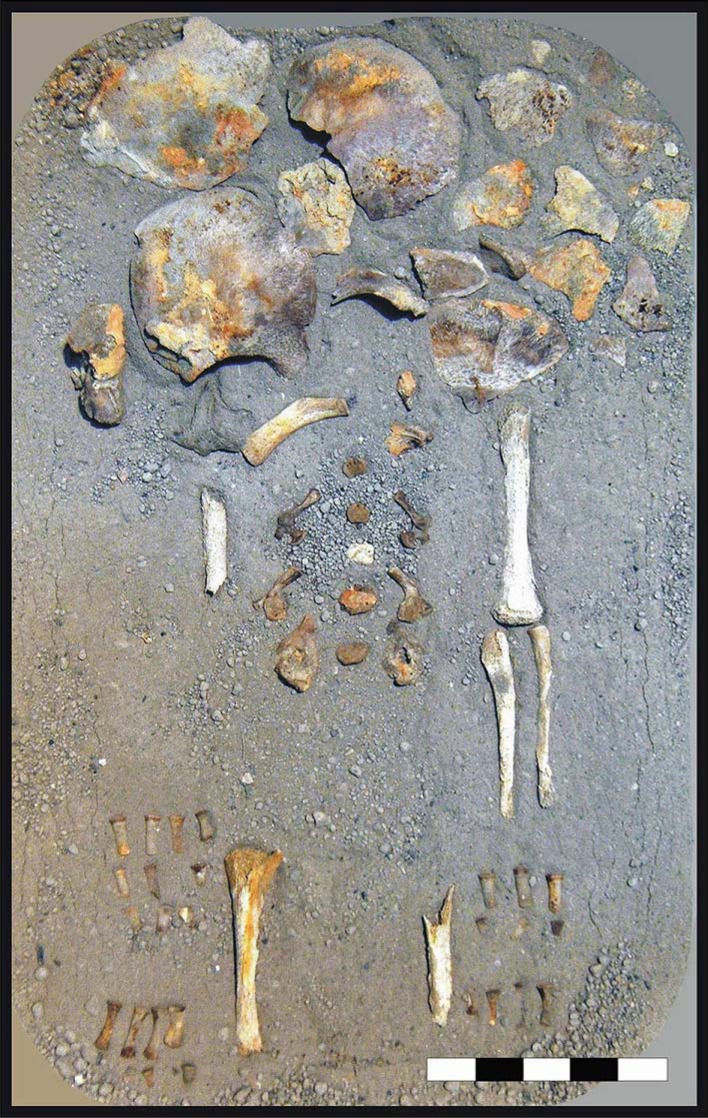
Thermal effects on a fetal skeleton. Intrauterine seven-month skeletal remains (specimen 15bis), unearthed from chamber 11. Spotted red residues, apparent on the skullcap surface, were found to be extremely rich in iron by ICP-MS analysis (55.587 mg/Kg), as well as those red residues (58.519 mg/Kg) encrusting the pelvis (sacrum) of the skeleton (specimen 15, adult female) within which they were found embedded (scale bar in cm).

New examination of the victims' skeletons and the taphonomic context now reveals the diffuse preservation of atypical red mineral residues encrusting the bones (Fig 7), which also impregnate the ash surge-bed below and around the corpses (Fig 8A and 8B), as well as the sand covering the chamber floor below the surge deposit (Fig 8C). Red mineral residues were also found to encrust both the skullcap (Fig 3A) and the inner table (Fig 3A, panel 1), and impregnate the ash filling the intracranial space as well (Fig 6A), mostly in the form of spotted inflorescence-shaped aggregates (Fig 6C). The above red residues were found in adults, children and some surprisingly well-preserved fetal skeletal remains (Fig 9), in the last case encrusting as well the adult pelvis bones from which they were unearthed. Similarly, black mineral residues may encrust both the outer and inner table (Fig 3B, panel 1 and 3C, panel 1), or laminate the ash filling the intracranial space (Fig 6B).

On the basis of color and location, the mineral residues were classified as: i. red incrustations; ii. black incrustations; iii. ash or sand impregnated by red residues; iv. ash or sand impregnated by black residues. In order to identify their chemical nature, we carried out a multi-element analysis [77,78] by means of inductively coupled plasma mass spectrometry (ICP-MS) [70,79]. We selected 103 samples (S2 Table) from the mineral residues either encrusting the bones and the intracranial space ranging in color from light red to dark red and black (Fig 7), or impregnating both the ash bed layers in which the skeletons were embedded and the sand bottom. For comparison, additional samples of ash from the ash-bed deposit and the sand bottom not in direct contact with the skeletons were also analyzed.

Results from ICP-MS analysis revealed an extremely high amount of iron in the class of red incrustations detected from the cranial and postcranial bones (C25, C27, C56, C69, C70, C72-C88, C90, C93-C102), the ash filling the skulls (C5, C26, C49, C50, C75, C83), the ash-bed (C7, C20, C30, C34, C35, C39, C44, C45, C103), and the sand (C22, C31, C46) (Table 1 and S4 Fig; see also S2 Table for type and source of samples). In contrast, samples of ash (S1 surge deposit) (C1-C3, C8, C9, C11, C12, C14, C53, C55, C58, C67) and sand (chamber bottom) (C10, C13, C37, C41, C42) non-impregnated by red residues showed an almost negligible amount of iron. The lowest values correspond to black incrustations from bones (C17, C18, C21, C23, C36, C38, C51, C91), ash (C19, C29) and sand (C28, C32, C41, C42). These findings indicate that the extremely high content of iron could not be ascribed to volcanic ash or other volcanic products, suggesting that it might have originated from the victims' body fluids.

We further investigated this question by searching for the possible presence of heme-containing protein residues by means of protein approaches. The red incrustation areas from several iron-containing samples were submitted to trypsin digestion after different extraction procedures, and the putative protein digests were analyzed by liquid chromatography-tandem mass spectrometry (LC-MS/MS) [72]. No peptides originating from tissue proteins could be detected in the samples. However, although sampling did not notch into the bone but only the incrustation was taken, in several cases we were able to detect a few reliable peptides from human collagen proteins alpha-1(I) and alpha-2(I) (data from sample C5 are reported in S3 Table as an example). These results indicate that only proteins protected by the inorganic scaffold of the bones survived the unbearable temperature experienced, while proteins unprotected by the hard tissues were totally destroyed.

We then addressed the matter by Raman investigation on selected and representative samples in order to identify or exclude various iron-containing compounds, and identify other possible non-ferrous species. In particular, we sought to detect and discriminate the possible preservation of heme and heme-related degradation products within the red and black mineral residues. In all, 22 samples were selected on the basis of iron content data provided by ICP-MS and analyzed using Raman microspectroscopy with 514 and 647 nm excitation (Table 2). Microscopically heterogeneous residues showing spots of different colors (13, 22, 46, 49, 51, 75 and 92) were sampled several times in different areas, typically red, dark reflecting and grey areas. Special attention was paid to possible heme or heme-degradation products [71]. Twelve samples did not provide any Raman band (5, 8, 27, 40, 44, 56, 73, 89, 99, 108, 144), while the remaining eleven samples showed Raman features. Among the latter, samples 22, 25, 46 and 49 taken from red encrusted material showed iron-related bands (Table 2), consistently with ICP-MS analysis. Raman analysis shows iron compounds also in samples 21 and 49, despite the low iron content measured via ICP-MS.

**Table 2:** Results obtained by Raman microspectroscopy. Last two columns refer to Raman traces in S5 Fig and to literature used for band assignment.

Sample Number	Chamber	Skeleton Number	Type of sample	Source	Fe (mg/kg)	Raman bands (cm-1)*	Parts in heterogeneous samples	Putative assignment	Label in S5 Fig.	Ref
C 5	10	26	red incrustations	endocranial ash cast	81037,8	no Raman feature detected	/	/		
C 8	10	/	ash S1	ash	1709,2	no Raman feature detected	/	/		
C 13						329 (m), 390 (m), 536 (m), 667 (s) 1007 (m), 1036 (m), 1364(m), 1603 (m)	dark red	pyroxene, carbon	C13 a	[81,84]
						282(m), 334(w), 404 (w), 473 (m), 514 (s), 767 (w), 805 (w), 1020 (w), 1125 (m), 1364 (w), 1600 (w)	dark	plagioclase, carbon	C13 b	[81]
C 21	12	22	black incrustations	endocranial cavity	302,0	217(w), 295(vw), 399(w), 500(m), 577(s), 655(sh), 722(sh), 1067(w), 1205(w), 1329(vw)	red	plagioclase, magnetite, hematite, carbonate	C21	[81]
C 22	12	5	sand + red incrustations	level ash/sand	75593,1	256(vw), 332(m), 514(w), 671(vs) 1015(vs)	dark reflecting surface	pyroxene	C22 a	[81]
						217 (vw), 306 (vw), 502 (s), 582 (m), 622 (w), 693 (s), 1061 (w), 1247 (w)	red spot	plagioclase, iron oxides, SiO2 glasses	C22 b	[81,83, 92]
						285 (w), 406 (w), 507 (w), 680 (m), 723 (w), 764 (vw), 1324 (w), 1583 (w)	dark	hematite, magnetite, carbon	C22 c	[80]
						500 (s) 530(m) 999 (vw), 1074 (w)	grey zone	plagioclase	C22 d	[81]
						285 (m), 407 (w), 476 /w), 512 (s), 672 (m)	dark red	plagioclase, magnetite	C22 e	[80,81]
C 25	10	19	red incrustations	femur + acetabulum	44370,0	359 (w), 500 (w) 706 (m), 1382 (w), 1607 (vw)	dark	maghemite, carbon	C25	[84,92]
C 27	11	15	red incrustations	coccyx female + fetus	55586,7	no Raman feature detected	/	/		
C 40	12	?	ash S1 + iron metal residues	ash from iron bracelet	21890,6	no Raman feature detected	/	/		
C 44	10	3	ash S1 + red incrustations	ileum (skeleton/ash)	31859,2	no Raman feature detected	/	/		
C 45	12	3	ash S1 + red incrustations	under foot (skeleton/ash)	32418,5	597 (m), 683 (w), 1370 (w), 1602 (m)	dark	carbon	C45	[84]
C 46	10	SP-2	sand + ashS1 + red incrustations	under pelvis/legs	30895,4	346 (w), 714 (m), 1388 (w), 1597 (w)	dark	maghemite, carbon	C46 a	[80,84]
						227 (w), 247 (w), 294 (m), 413 (m), 515 (m), 611 (w), 666 (w), 1324 (m)	red	hematite	C46 b	[81]
						327 (m), 393 (m), 559 (w), 667 (m), 770 (vw), 817 (vw), 1010 (s)	black	pyroxene	C46 c	[81]
C 49	12	24	red incrustations	endocranial ash cast	71563,1	419 (w), 499 (w), 585 (w), 623 (w), 673 (w), 1013 (s) 1141 (w)	dark	gypsum, magnetite	C49 a	
						291 (w), 480 (m), 520 (s), 663 (w) 967 (vw)	black	plagioclase, magnetite, apatite	C49 b	[81]
						210 (vw), 505 (w), 593 (w), 668 (m), 1138 (w), 1366 (m), 1580 (s)	black	carbon, iron oxides, SiO2 glasses	C49 c	[83,84]
C 50	11	15bis	red incrustations	7m fetal skull	58519,0	no Raman feature detected	/	/		
C 51	10	11A	black incrustations	sacrum	411,7	210 (vw), 403 (vw), 504 (m), 576 (m), 671 (s), 1064 (w), 1218 (w), 1349 (vw)	reddish	plagioclase, carbonate, iron oxides, SiO2 glasses	C51 a	[81,83]
						395 (w), 576 (w), 666 (m), 1009 (m)	black	pyroxene	C51 b	[81]
						280 (w), 1088 (m)		CaCO3	C51 c	[81]
						617 (br), 503 (w)	red	iron oxides, SiO2 glasses	C51 d	[83,92]
C 56	5	3	red incrustations	teeth	18695,6	no Raman feature detected	/	/		
C 73	10	Sp-4 Q2b	red incrustations	rib	19456,3	no Raman feature detected	/	/		
C 75	10	Sp-6 Q2b-1c	red incrustations	skull	86945,4	963	dark	Apatite	C75 a	
						301 (w), 511 (w), 670 (m), 964 (vw)	dark red	magnetite, plagioclase, apatite	C75 b	
C 89	10	10	red material	cloth tissue?	846,0	No Raman feature detected	/	/		
C 92	10	17	black + red incrustations	scapula + clavicle	737,0	227 (w), 297 (m), 412 (w), 512 (m), 575 (w), 619 (vw), 680 (s), 1329 (m), 1594 (w)	red	hematite, magnetite, carbon	C92 a	[84]
						502(m) 580(s) 663(s), 1360 (vw) 1593(w)	black	carbon, iron oxides, SiO2 glasses	C92 b	[84]
C 99	12	16	red incrustations	rib + sternum	76455,3	no Raman feature detected	/	/		

*s (strong), m (medium), w (weak), sh (shoulder), br (broad), vs (very strong), vw (very weak).

## Discussion

Raman investigation (Table 2 and S5 Fig) showed both chemical compounds typical of volcanic ashes (SiO_2_ glass, plagioclase, pyroxenes) and other compounds, compatible with human body degradation (carbon and several iron oxides), frequently coexisting (samples 22, 25, 46, 49, 92). Particularly, Raman investigation of red spots from many encrusted samples clearly showed bands characteristic of iron-containing compounds such as hematite, magnetite and maghemite (samples 21, 22, 25, 46, 49, 75, 92 in Table 2 and S5B and S5B Fig) [80]. A possible iron-containing carbonate (hydrotalcite) was sporadically detected in sample 21, consistently with Raman analysis of other volcanic eruptions [81], while no Raman band from pyrite or ZnS with Fe excess [82], iron carbonate siderite or iron sulphate jarosite [81] were detected. Large envelope of Raman bands in the region 500–700 cm^-1^ (in C21, C22b, C51a, C51d, C92a, C92b) can be assigned to a mixture of poorly crystalline iron oxides, along with SiO_2_ glasses [83]. Dark spots in incrustations frequently showed other crystalline materials from ashes, such as pyroxene (in samples 13, 22, 46, 51) and plagioclase (in samples 2, 13, 49 and 75) [81]. Many dark spots showed carbon-related D and G bands at 1600 and 1350 cm^-1^ (C13a, C13b, C22c, C25, C45, C46a, C49c, C92c) [84]. Bands related to gypsum (or less likely related to SO_2_ inclusion [81]) were observed in sample 49a. No CO_2_, H_2_O and H_2_S inclusions, observed of Vesuvius magma [85], were detected in this study.

The possible implications of the above Raman results to the fluid-body degradation is below discussed. Regarding the origin of the observed iron oxides (from mineral or from body fluids), indeed we have no direct indication. We can only find indirect evidence of compatibility with an organic origin.

Heme degradation has been extensively studied in the temperature range of meat cooking (70–100°C). Already at such temperatures heme partially degrades, releasing iron without any porphyrin cleavage [86–88]. At higher temperature (400°C), iron-free porphyrins undergo a two-stage degradation of the methylene bridges [89] and metallo-porphyrins also undergo cleavage reaction [90], generating anhydrous metal oxides as final products [91].

So, the expected heme-degradation products from victims of Vesuvius are only iron oxides. In our study hematite was both observed alone in some samples (C22c, C46b, C92c) and in combination with other iron oxides (e.g. magnetite in C51b). It is worth reminding that multiple mechanisms of interconversion of iron oxides occur as a function of temperature and oxidizing environment [92], so heme degradation is compatible with observations of multiple iron oxides. Furthermore, the coexistence of amorphous carbon (most likely product of organic combustion) and iron oxides with broad bands (thus not very crystalline) on the same samples (22, 25, 46, 49, 92) can well be compatible, if not in support, with residues of human body source, rather than simply related to pumice Vesuvian composition [85].

Recent multidisciplinary research on the lethal effects of the pyroclastic surges induced by the 79 AD eruption in the Vesuvius area showed that in the vicinity of Pompeii heat was the main cause of death of those who had previously been thought to have died of ash suffocation [41]. This was also posited for the victims of Herculaneum, specifically for those who had taken refuge in waterfront chambers along the beach and were then sheltered from direct mechanical impact, but not from heat of the emplacing S1 surge [34,39–43].

In the present work, careful inspection of the victims' skeletons revealed cracking and explosion of the skullcap and blackening of the outer and inner table, associated with black exudations from the skull openings and the fractured bone. Such effects appear to be the combined result of direct exposure to heat and an increase in intracranial steam pressure induced by brain ebullition, with skull explosion as the possible outcome [93]. As the final result of the victims’ bodies being engulfed by the hot pyroclastic surge, the intracranial cavity is found to be filled by ash in the form of a brain-like ash cast. Experimental research shows black bone to be indicative of high thermal exposure (ca 500 °C) [29,41] even if coexistence of blackened bone and bone of unchanged color suggests the persistence of a cooler intracranial area possibly due to temporary preservation (prior to definitive vanishing) of a residual brain mass, since intense heat forces the dural layers to shrink, which in turn constricts the brain into a dense mass [93].

As to the effects of exposure to thermal destruction, a huge literature is available [54,65–68,79,93–96]. As to the finding of dark stained bone at Herculaneum, bone black in color represents carbonized skeletal material in direct contact with heat or flames [97,98]. Dark colors, particularly black, are related to the carbonization of collagen [64,99]. Heating can be related to combustion (with oxygen) or charring (without oxygen), both of which require the formation of char [100]. The heating process depends also on temperature, heating rate (C°/min) and exposure time [101].

At Herculaneum, the direct contact of the soft tissues with the pyroclastic surge indicates that the charring was caused by hot-emplaced volcanic ash [102], a characteristic uncommon for victims of pyroclastic density currents, whose bodies are mostly preserved [48,103]. In the 79 AD eruption, assuming environmental reducing conditions (lack or low content of oxygen) at the surge emplacement [6,48], the dark staining of bones is likely to be due to a charring process affecting the victims engulfed within the hot ash cloud [104]. This particular condition seems confirmed by the results of experimentally heated bone vs victims’ bones from the 79 AD volcanic context [41,100]. The soft tissues of a corpse act as a physical barrier against the heat and keep bones in anaerobic conditions [105]. The latter process and the unevenness of soft tissue thickness in the body and an unequal distribution of heat during exposure itself, possibly due to the different corpses distribution in the chambers (S1C Fig), may explain the difference in color alterations and the varying degrees of charred bones in the same individual or even on a single bone [106–108].

With regard to heat-induced effects on the skull, bone can display a sequence of charred, border and heat line zones which define the area of bone exposure to heat. As also detected in forensic cases [98,109], the changes in the visual appearance of thermally altered bone result in a scale that gradually evolves from a translucent yellow (unaltered bone) to an opaque white (heat line and border), to a blackened appearance (char). The particular evidence of skullcap fractures and sutures characterized by strikingly black staining has been interpreted as openings for fluids to vent from the brain case. This “venting” is said to trap fluids and tissue on the surface of the bone, causing it to be imbued black [110].

As regards the thermal origin of cracking detected on the skull of the 79 AD eruption victims, heat-induced fractures are always limited to the charred areas, since developing heat fractures do not have the energy to radiate out of charred areas into the uncharred bone [98]. This evidence is particularly significant since demonstrates the perimortem origin of the skull fractures induced by the hot ash surge, excluding postmortem causes like the weight of the ash deposit or the direct impact of the surge itself, as previously hypothesized [28,34]. In such cases, the skeletons would have been at least partly dismembered or crushed, which is not, as demonstrated by complete preservation of the victims’ skeletons and their anatomical joint connection (Fig 1).

As to the mechanism of death at Herculaneum, evidence like the red residues rich in iron oxides detected from the ash filling the intracranial cavity and encrusting the inner and the outer table, as well as the brown coloration of the venous sinuses, strongly suggests massive heat-induced hemorrhage [111] and a rise in intracranial pressure, as appears clearly from recurrent skull explosive fracture [94]. In forensic cases of skull bursting, particularly in children, the expelled brain matter may form a circular pattern around the head [93], a feature also occurring in a few Herculaneum children (Fig 3A). Examination of fire victims has also shown the presence of heat hematoma [112], with brown bone color being associated with hemoglobin [94]. This is a heat-induced coagulation lying between the bone and the dura, caused by exudation from the venous sinuses of boiling blood, which becomes spongy and brown. The bone table overlying the hematoma is usually charred [113], as repeatedly seen in the victims' skulls at Herculaneum. An increase in pressure caused by bleeding in the various compartments of the brain is considered the most common mechanism of sudden death [95].

Evidence of a heat-induced process of rapid body flesh disappearing is given by the incipient "pugilistic attitude" testified by rare flexure of the upper limbs, but not yet evident in the lower ones (Fig 1A). This heat-induced posture results from denaturation of proteins and muscle fiber dehydration which cause rapid muscle contraction, with consequent abduction of the limbs to the body [94]. Since a body shows a pugilistic attitude soon after exposure to pyroclastic surge temperatures of around 200°C to 250°C [48] or burning for about 10 minutes in a crematorium at temperatures between 670°C and 810°C [96], the lack of a complete pugilistic pose in the victims' corpses at Herculaneum may indicate that the muscles disappeared more quickly than they contracted. This also seems attested by the "life-like" stance observed in the victims' corpses resulting from the extraordinarily well-preserved skeletal joints fixing the body shape in three-dimensional space (Fig 1B), that could only be explained by very rapid replacement of flesh by ash. In contrast, the widespread occurrence of a pugilistic attitude in the Pompeii victims is attributable to the long-lasting persistence of body flesh, apparent from the shape of the plaster casts, as a consequence of exposure to a lower temperature estimated to be around 250–300°C [41], enough to cause muscle contraction but insufficient for soft tissues to vanish rapidly.

## Conclusions

Here we show for the first time convincing experimental evidence suggesting the rapid vaporization of body fluids and soft tissues of the 79 AD Herculaneum victims at death by exposure to extreme heat, as testified by the unique preservation of iron and heme-iron degradation products as a result of thermally induced hemoprotein oxidation and denaturation. The occurrence of unexpectedly very high concentrations of iron was detected by ICP-MS analysis in samples showing red incrustations from bones, volcanic ash and sand compared to those unaffected, which might have originated from body fluids. Raman micro spectroscopic investigation of these samples reveals the presence of many iron-containing inorganic compounds, including several iron oxides, expected to be the final products of heme-iron upon thermal decomposition. The detection of such iron-containing compounds from the skull and the ash filling the endocranial cavity, coupled with brown coloration of venous sinuses, bone blackening and cracking, strongly suggests a widespread pattern of heat-induced hemorrhage, intracranial pressure increase and bursting, most likely to be the cause of instant death of the inhabitants in Herculaneum.

These findings highlight the need for thorough evaluation of key bioanthropological and taphonomic evidence during archaeological investigations. This is particularly true for the sites affected by the 79 AD Vesuvius eruption, given the high-risk scenario for three million people living today close to the volcano, even if sheltered within buildings.

## Supporting information

S1 Fig The Roman town of Herculaneum. A. Map showing approximate location of the site; B. View of the archaeological excavations; C. The seafront chambers crowded with different numbers of victims’ skeletons; D. A group of victims unearthed in chamber 12; E. Planimetry of chamber 10 (The authors created the image themselves. Photos [B, D] and images [C, E] by P. Petrone).
(DOCX)

S2 Fig **Planimetric sections of the town (A) and a boat-chamber (B).** A. Section of the inferior IV *cardo*, the suburban area and the chambers facing the sea. Note the turbulent surge cloud passing through the town and emplacing on the beach and within the chambers; B. Section of the seafront area with the victims buried within the ash surge deposit (images modified from Budetta, 1993 and Sigurdsson et al., 1985).
(DOCX)

S3 Fig Thermal microscopic and ultra-structural modifications in human victims' bones from Herculaneum. Adult bone analyzed with a light microscope (scale bar 100 μm) and a scanning electron microscope (scale bar 10 μm, 1700×): Radius showing both linear and polygonal cracking (A) and incipient recrystallization (B). At 500–800°C the basic bone structure recrystallizes into irregular globules (17).
(DOCX)

S4 Fig Iron amounts resulting from inductively coupled plasma mass spectrometry performed on 103 specimens. (DOCX)

S5 Fig Representative Raman spectra. A. Samples 13, 21, 22, 25 and 45. Ap (apatite), C (amorphous carbon), Ca (Carbonate), He (hematite), Gl (SiO_2_ glasses), Io (mixture of multiple iron oxides), Ih (iron oxyhydroxides), Ma (Magnetite), Mg (maghemite), Pl (Plagioclase), Py (Pyroxene); B. samples 46, 49, 51, 75, 92. Ap (apatite), C (amorphous carbon), Ca (Carbonate), Gl (SiO_2_ glasses), Gy (gypsum), He (hematite), Io (mixture of multiple iron oxides), Ma (Magnetite), Mg (maghemite), Pl (Plagioclase), Py (Pyroxene). Frequencies have been reported in Table 2.
(DOCX)

S1 Table Sex and average age at death of the 80 victims' skeletons found in chambers 5, 10, 11, and 12 in 1997–1999 archaeological investigations in the suburban area of Herculaneum. Individual A was one of the very few victims found in the town.
(DOCX)

S2 Table Type of samples analyzed and their source. (DOCX)

S3 Table Details of the identification of human collagen proteins alpha-1(I) and alpha-2(I) in sample C5. (DOCX)

## References

[1] Mastrolorenzo G, Petrone P, Pappalardo L, Sheridan MF. The Avellino 3780 yr B.P. catastrophe as a worst-case scenario for a future eruption at Vesuvius. Proc Natl Acad Sci USA. 2006; 103: 4366–4370. 10.1073/pnas.0508697103 16537390 PMC1450177

[2] Sheridan MF, Barberi F, Rosi M, Santacroce R. A model for plinian eruption of Vesuvio. Nature. 1981; 289: 282–285.

[3] Lockwood JP, Hazlett RW. Volcanoes: Global Perspectives Oxford: Wiley-Blackwell; 2010.

[4] NASA Earth Observatory. Mount Vesuvius, Naples, Italy: Image of the Day. 2006. Available from: https://earthobservatory.nasa.gov/IOTD/view.php?id=6403

[5] Petrone P. Human corpses as time capsules: New perspectives in the study of past mass disasters. J Anthropol Sci. 2012; 89: 3–6. 10.4436/jass.89008 21730365

[6] Sigurdsson H, Carey S, Cornell W, Pescatore T. The eruption of Vesuvius in A.D. 79. Nat Geogr Res. 1985; 1: 332–387.

[7] Barberi F, Macedonio G, Pareschi MT, Santacroce R. Mapping the tephra fallout risk: An example from Vesuvius, Italy. Nature. 1990; 344: 142–144.

[8] Sigurdsson H, Cashdollar S, Sparks RSJ. The eruption of Vesuvius in A.D. 79: Reconstruction from historical and volcanological evidence. Am J Archeol. 1982; 86: 39–51.

[9] Giacomelli L, Perrotta A, Scandone R, Scarpati C. The eruption of Vesuvius of 79 AD and its impact on human environment in Pompeii. Episodes. 2003; 26: 234–237.

[10] Luongo G, Perrotta A, Scarpati C. Impact of the AD 79 explosive eruption on Pompeii, I. Relations amongst the depositional mechanisms of the pyroclastic products, the framework of the buildings and the associated destructive events. J Volcanol Geotherm Res. 2003a; 126: 201–223.

[11] Maiuri A. Herculaneum. Rome; 13, 1977.

[12] Maiuri A. Ercolano. I nuovi scavi (1927–1958), Roma, 1958.

[13] Maiuri A. Pompeii. Scientific American. 1958; 198: 70.

[14] Luongo G, Perrotta A, Scarpati C, De Carolis E, Patricelli G, Ciarallo A. Impact of the AD 79 explosive eruption on Pompeii, II. Causes of death of the inhabitants inferred by stratigraphical and areal distribution of the human corpses. J Volcanol Geotherm Res. 2003b; 126: 169–200.

[15] Thomas ML. Oplontis B: a center for the distribution and export of Vesuvian wine. Journal of Roman Archaeology. 2015; 28: 403–411.

[16] Nunziante L, Fraldi M, Lirer L, Petrosino P, Scotellaro S, Cicirelli C. Risk assessment of the impact of pyroclastic currents on the towns located around Vesuvio: A non-linear structural inverse analysis. Bull Volcanol. 2003 65: 547–561.

[17] Fergola L. Oplontis In: D’Ambrosio A, Guzzo PG, Mastroroberto M editors. Storie da un’eruzione. Pompei Ercolano Oplontis. Milano, Italy, pp. 152–157; 2003 (Italian).

[18] Dobran F, Neri A, Todesco M. Assessing the pyroclastic flow hazard at Vesuvius. Nature. 1994; 367: 551–554.

[19] De Carolis E, Patricelli G. Rinvenimenti di corpi umani nel suburbio pompeiano e nei siti di Ercolano e Stabia. Rivista di Studi Pompeiani. 2013a; 24: 11–32 (Italian).

[20] Mastrolorenzo G, Pappalardo L, Ricciardi I, Petrone PP. Active volcanism and related events in Campania: Primary and secondary effects of explosive volcanic eruptions on the environment and people. 32nd International Geological Congress, Florence, Italy; Post-Congress P67, Vol 6, pp. P55-PW06; 2004. Available from: http://www.isprambiente.gov.it/files/pubblicazioni/periodicitecnici/memorie/memorielxiii/p67.pdf

[21] De Carolis E, Patricelli G. Le vittime dell’eruzione In: D’Ambrosio A, Guzzo PG, Mastroroberto M editors. Storie da un’eruzione. Pompei Ercolano Oplontis. Milano, Italy, pp. 56–72; 2003b (Italian).

[22] Ruggiero M. Degli scavi di Stabia dal MDCCXLIX al MDCCLXXXII, Napoli, 1881(Italian).

[23] Gore R, Mazzatenta OL. After 2000 years of silente, the dead do tell tales at Vesuvius. Natl Geogr. 1984; 165: 557–613.

[24] Bisel SC. Human bones at Herculaneum. Riv. Studi Pompeiani. 1987; I: 123–131.

[25] Bisel SC. Nutrition in first century Herculaneum. Anthropologie. 1988; 26: 61–66.

[26] Bisel SC. The human skeletons of Herculaneum. Int J Anthropol. 1991; 6: 1–20.

[27] Capasso L. Herculaneum victims of the volcanic eruptions of Vesuvius in 79 AD. The Lancet. 2000; 356: 1344–1346. 10.1016/S0140-6736(00)02827-0 11073037

[28] Capasso L. I fuggiaschi di Ercolano Paleobiologia delle vittime dell’eruzione vesuviana del 79 d.C. Roma: L’Erma di Bretschneider, 2001 (Italian).

[29] Capasso L. Bacteria in two-millennia-old cheese, and related epizoonoses in Roman populations. J Infection. 2002; 45: 122–127. 10.1053/jinf.2002.0996 12217720

[30] Capasso L, Di Tota. Lice buried under the ashes of Herculaneum. The Lancet. 1998; 351: 992. 9734976

[31] Capasso L, Capasso L. Mortality in Herculaneum before volcanic eruption in 79 AD. The Lancet. 1999; 354: 1826. 10.1016/S0140-6736(05)70601-2 10577678

[32] Capasso L, Capasso L, Caramiello S, D'Anastasio R, Di Domenicantonio L, Di Fabrizio A et al Paleobiology in the population of Herculaneum (79 AD). Recenti Prog Med. 2000; 91: 288–296. 11512386

[33] Ottini L, Di Tota G, Mariani-Costantini R, Angeletti L, La Verghetta M, Capasso L et al Evidence of a forearm fracture in a young victim of the 79 AD Vesuvius eruption. J Paleopathol. 2002; 13: 23–26.

[34] Shmidt CW, Oakley E, D'Anastasio R, Brower R, Remy A, Viciano J. Herculaneum In: Schmidt CW, Symes SA editors. The analysis of burned human remains. Academic Press 2015, pp. 149–161.

[35] Torino M, Rognini M, Fornaciari G. Dental fluorosis in ancient Herculaneum. The Lancet. 1995; 345: 1306. 10.1016/s0140-6736(95)90952-4 7746075

[36] Budetta T. I nuovi scavi nell’area suburbana di Ercolano. In: Ercolano 1738–1988. 250 anni di ricerca archeologica. Roma, 1993, pp. 677–690 (Italian).

[37] Petrone P. Le vittime dell’eruzione del 79 AD. In: Storie da un’eruzione: in margine alla mostra. Napoli, 2005, pp. 31–44 (Italian).

[38] Petrone P. Dallo scavo al museo: uomini e vulcani. Museologia Scientifica Memorie. 2011; 8: 178–183 (Italian).

[39] Petrone P, Fedele F (editors). Vesuvio 79 A.D. Vita e morte ad Ercolano Napoli: Fridericiana Editrice Universitaria, 2002 (Italian).

[40] Mastrolorenzo G, Petrone P, Pagano M, Incoronato A, Baxter PJ, Canzanella A et al The Herculaneum victims of Vesuvius in A.D. 79. Nature. 2001a; 410: 769–770. 10.1038/35071167 11298433

[41] Mastrolorenzo G, Petrone PP, Pappalardo L, Guarino FM. Lethal thermal impact at the periphery of pyroclastic surges: evidences at Pompeii. PloS ONE. 2010; 5(6) e11127/1–12, 10.1371/journal.pone.0011127 20559555 PMC2886100

[42] Mastrolorenzo G, Petrone P, Pagano M, Incoronato A, Baxter PJ, Canzanella A et al The 79 AD Vesuvius plinian eruption at Herculaneum and its impact on the people Juvigné E, Raynal JP editors. In: Tephras. Chronology, Archaeology. Goudet: CDERAD éditeur 2001b; 183–189.

[43] Petrone P, Niola M, Di Lorenzo P, Graziano V, Paternoster M, Buccelli C. A New Forensic Approach to Past Mass Disasters: The Human Victims of Vesuvius. Austin J Forensic Sci Criminol. 2014; 1: 1–2.

[44] Petrone PP, Giordano M, Giustino S, Guarino FM. Enduring Fluoride Health Hazard for the Vesuvius Area Population: The Case of AD 79 Herculaneum, Plos ONE. 2011; 6(6): e21085 10.1371/journal.pone.0021085 21698155 PMC3116870

[45] Petrone PP, Guarino FM, Giustino S, Gombos F. Ancient and recent evidence of endemic fluorosis in the Naples area. J Geochem Explor. 2013; 131: 14–27.

[46] Guarino FM, Buccelli C, Graziano V, La Porta P, Mezzasalma M, Odierna G et al Recovery and amplification of ancient DNA from Herculaneum victims killed by the 79 AD Vesuvius hot surges. Turk J Biol. 2017; 41: 640–648.

[47] Martyn REV, Garnsey P, Fattore L, Petrone P, Sperduti A, Bondili L et al Capturing Roman dietary variability in the catastrophic death assemblage at Herculaneum. J Archaeol Sci Rep. 2018; 10.1016/j.jasrep.2017.08.008

[48] Baxter P. Medical effects of volcanic eruptions: I Main causes of death and injury. Bull Volcanol. 1990; 52: 532–544.

[49] Hansell AL, Horwell CJ, Oppenheimer C. The health hazards of volcanoes and geothermal areas. Occup Environ Med. 2006; 63: 149–156. 10.1136/oem.2005.022459 16421396 PMC2078062

[50] Baxter PJ, Boyle R, Cole P, Neri A, Spence R, Zuccaro G. The impacts of pyroclastic surges on buildings at the eruption of the Soufrière Hills volcano, Montserrat. Bull Volcanol. 2005; 67: 292–313.

[51] Druitt TH. Pyroclastic density currents. In: Gilbert GS, Sparks RSJ editors. The physics of explosive volcanic eruptions. Geological Society of London Special Publication. 1998: 145: 145–182.

[52] Auker M, Sparks R, Siebert L, Crosweller H, Ewert J. A statistical analysis of the global historical volcanic fatalities record. J Appl Volcanol. 2013; 2: 1–24.

[53] Baxter PJ, Aspinall W, Neri A, Zuccaro G, Spence R, Cioni R et al Emergency planning and mitigation at Vesuvius: a new evidence-based approach. J Volcanol Geotherm Res. 2008; 178: 454–73.

[54] Baxter PJ, Jenkins S, Seswandhana R, Komorowski J-C, Dunn K, Purser DA et al Human survival in volcanic eruptions: Thermal injuries in pyroclastic surges, their causes, prognosis and emergency management. Burns. 2017; (in press). 10.1016/j.burns.2017.01.025 28233579

[55] Kent DV, Dragoslav N, Pescatore T, Sparks SRJ. Paleomagnetic determination of emplacement temperature of Vesuvius A.D. 79 pyroclastic deposits. Nature. 1981; 290: 393–396.

[56] Incoronato A, Spina G, Mastrolorenzo G, Pagano M. Determinazione delle temperature di deposizione, mediante procedure paleomagnetiche, di piroclastiti del 79 d.C. ad Ercolano In: D’Amico C, Arbore Livadie C (a cura di), Le Scienze della Terra e l’Archeometria. Napoli: Centre Jean Bérard 1997; pp. 95–98.

[57] Cioni R, Gurioli L, Lanza R, Zanella E. Temperatures of the A.D. 79 pyroclastic density current deposits (Vesuvius, Italy). J Geophys Res. 2004; 109: 1–18. B02207 10.1029/2002JB002251

[58] Giordano G, Zanella E, Trolese M, Baffioni C, Vona A, Caricchi C et al Thermal interactions of the AD79 Vesuvius pyroclastic density currents and their deposits at Villa dei Papiri (Herculaneum archaeological site, Italy). Earth Plan Sci Let. 2018; 490: 180–192.

[59] Caricchi C, Vona A, Corrado S, Giordano G, Romano C. AD 79 Vesuvius PDC deposits’ temperatures inferred from optical analysis on woods charred in-situ in the Villa dei Papiri at Herculaneum (Italy). J Volcanol Geotherm Res. 2014; 289: 14–25.

[60] Sparks RSJ. Grain size variation in ignimbrites and implications for the transport of pyroclastic flows. Sedimentology. 1976; 23: 147–188.

[61] Allison PA, Briggs DEG editors. Taphonomy: Releasing the data locked in the fossil record New York: Plenum Press 1991; pp. 25–69.

[62] Maiuri A. Last moments of the Pompeians. Natl Geogr. 1961; 120: 651–669.

[63] LaMotte RH, Campbell JN. Comparison of responses of warm and nociceptive C-fiber afferents in monkey with human judgments of thermal pain. J Neurophysiol. 1978; 41: 509–528. 10.1152/jn.1978.41.2.509 418156

[64] Shipman P, Foster G, Schoeninger M. Burnt Bones and Teeth: an Experimental Study of Color, Morphology, Crystal Structure and Shrinkage. J Archaeol Sci. 1984; 11: 307–325. 10.1016/0305–4403(84)90013–X

[65] Fernández Castillo R, Ubelaker DH, Acosta JA, de la Rosa RJ, Garcia IG. Effect of temperature on bone tissue: histological changes. For Sci Int. 2013; 226: 33–37. 10.1111/1556-4029.12093 23458344

[66] Brinkmann B, Kleiber M, Koops E, Püschel K. Vitale reaktionen bei akutem verbrühungstod. Z Rechtsmed. 1979; 83: 1–16. 442830

[67] Knűsel CJ, Janaway RC, King SE. Death, decay and ritual reconstruction: archaeological evidence of cadaveric spasm. Oxford J Archaeol. 2007; 2: 121–128.

[68] Camps FE, Robinson AE, Lucas BGB. Gradwohl’s Legal Medicine 3rd ed Bristol: John Wright & Sons Ltd; 1976.

[69] D’Ambrosio A, Guzzo PG, Mastroroberto M. (editors) Storie da un’eruzione. Pompei, Ercolano, Oplontis Milano: Electa 2003; pp. 526 (Italian).

[70] EPA. Method 6020B: Inductively Coupled Plasma—Mass Spectrometry, part of a Test Method for Evaluating Solid Waste Physical/Chemical Methods, SW-846 Update V, pp. 1–33; 2014.

[71] Neugebauer U, März A, Henkel T, Schmitt M, Popp J. Spectroscopic detection and quantification of heme and heme degradation products. Anal Bioanal Chem. 2012; 404: 2819–2829. 10.1007/s00216-012-6288-9 22903430

[72] Vinciguerra R, De Chiaro A, Pucci P, Marino G, Birolo L. Proteomic strategies for cultural heritage: form bones to paintings. Microchem J. 2016; 126: 341–348.

[73] Munsell Soil Color Charts. Baltimore, Md.: Munsell Color Company Inc.; 1954.

[74] Ellingham STD, Thompson TJU, Islam M, Taylor G. Estimating temperature exposure of burnt bone–A methodological review. Science and Justice. 2015: 55: 181–188. 10.1016/j.scijus.2014.12.002 25934370

[75] Nicholson RA. A morphological investigation of burnt animal bone and an evaluation of its utility in archaeology. J Archaeol Sci. 1993; 20: 411–428.

[76] Walker PL, Miller KWP, Richman R. 7—time, temperature, and oxygen availability: an experimental study of the effects of environmental conditions on the color and organic content of cremated bone In: Schmidt CW, Symes SA editors. The Analysis of Burned Human Remains, San Diego CA: Academic Press 2008; p. 129–135.

[77] Wilson CA, Davidson DA, Cresser MS. Multi-element soil analysis: an assessment of its potential as an aid to archaeological interpretation. J Archaeol Sci. 2008; 35: 412–424.

[78] Rasmussen KL, Tenorio AL, Bonaduce I, Colombini MP, Birolo L, Galano E et al The constituents of the ink from a Qumran inkwell: new prospects for provenancing the ink on the Dead Sea Scrolls. J Archaeol Sci. 2012; 39: 2956–2968.

[79] Schmidt CW, Symes SA. The analysis of burned human remains London: Elsevier; 2015.

[80] Das S, Hendry MJ. Application of Raman spectroscopy to identify iron minerals commonly found in mine wastes. Chem Geol. 2011; 290: 101–108.

[81] Lalla EA, López-Reyes G, Sansano A, Sanz-Arranz A, Martinez-Frías J, Medina J et al Raman IR vibrational and XRD characterization of ancient and modern mineralogy from volcanic eruption in Tenerife Island: Implication for Mars. Geosci Front. 2016; 7: 673–681.

[82] Hope GA, Woods R, Munce CG. Raman microprobe mineral identification. Min Eng. 2001; 14: 1565–1577.

[83] Ivleva NP, Huckele S, Weinzierl B, Niessner R, Haisch C, Baumann T. Identification and characterization of individual airborne volcanic ash particles by Raman microspectroscopy. Anal Bioanal Chem. 2013; 405: 9071–9084. 10.1007/s00216-013-7328-9 24121468

[84] Jehlicˇka J, Urban O, Pokorny J. Raman spectroscopy of carbon and solid bitumens in sedimentary and metamorphic rocks. Spectrochimica Acta Part A. 2003; 59: 2341–2352. 10.1016/s1386-1425(03)00077-5 12909147

[85] De Vivo B, Lima A, Webster JD. Volatiles in magnetic volcanic systems. 2005; 1: 19–24.

[86] Igene JO, King JA, Pearson AM, Gray JI. Influence of heme pigments, nitrite, and non-heme iron in development of warmed-over flavour (WOF) in cooked meat. J Agric Food Chem. 1979; 27: 838.

[87] Schricker BR, Miller DD. Effect of cooking and chemical treatment on heme and nonheme iron in meat. J Food Sci. 1983; 48: 1340.

[88] Chen CC, Pearson AM, Gray JI, Fooladi MH, Ku PK. Some factors influencing the non-heme iron content of meat and its implications in oxidation. J Food Sci. 1984; 49: 581.

[89] Guan C, Li L, Chen D, Gao Z, Sun W. Thermal behaviour and thermal decomposition study of porphyrin polymers containing different spacer groups. Thermochim Acta. 2004; 413: 31.

[90] O’Carra P. Heme cleavage: biological systems and chemical analogs In: Smith KE, editor. Porphyrins and metalloporphyrins. Amsterdam: Elsevier; 1975.

[91] Antina EV, Balantseva E V, Berezin MB. Oxidative Degradation of Porphyrins and Metalloporphyrins under Polythermal Conditions. Russian J Gen Chem. 2011; 81: 1222–1230.

[92] de Faria DLA, Venaüncio Silva S, de Oliveira MT. Raman Microspectroscopy of Some Iron Oxides and Oxyhydroxides. J Raman Spectr. 1997; 28: 873–878.

[93] Knight B. Forensic Pathology London: Edward Arnold; 1991.

[94] Saukko P, Knight B. Knight's Forensic Pathology London: Edward Arnold Ltd; 2004.

[95] Bohnert M, Rost T, Pollak S. The degree of destruction of human bodies in relation to the duration of the fire. For Sci Int. 1998; 95: 11–21. 10.1016/s0379-0738(98)00076-0 9718667

[96] Redsicker DR, O'Connor JJ. Practical Fire and Arson Investigation New York: Elsevier; 1997.

[97] Hermann B. Anthropologische bearbeitung der leichenbranden von Berlin-Rudow. Ausgrab. Berlin. 1970; 1: 61–71

[98] Symes SA, Rainwater CW, Chapman EN, Gipson DR, Piper AL. Patterned thermal destruction in a forensic setting In: Schmidt CW, Symes SA editors. The analysis of burned human remains. London: Academic Press 2008; pp. 15–54.

[99] Symes SA, L’Abbé EN, Stull KE, LaCroix M, Pokines JT. Taphonomy and the timing of bone fractures in trauma analysis In: Pokines JT, Symes SA editors. Manual of forensic taphonomy. Boca Raton FL: CRC Press. 2014; pp. 341–366.

[100] Reidsma FH, van Hoesel A, van Os BJH, Megens L, Braadbaart F. Charred bone: Physical and chemical changes during laboratory simulated heating under reducing conditions and its relevance for the study of fire use in archaeology. J Archaeol Sci Rep. 2016; 10: 282–292.

[101] Braadbaart F, Wright PJ, van der Horst J, Boon JJ. A laboratory simulation of the carbonization of sunflower achenes and seeds. J Anal Appl Pyrolysis. 2007; 78: 316–327.

[102] Jiang B, Harlow GE, Wohletz K, Zhou Z, Meng J. New evidence suggests pyroclastic flows are responsible for the remarkable preservation of the Jheol biota. Nature Comm. 2014; 5:3151 10.1038/ncomms4151 24495913

[103] Scott AC, Sparks RSJ, Bull ID, Knicker H, Evershed RP. Temperature proxy data and its significance for the understanding of pyroclastic density currents. Geology. 2008; 36:143–146.

[104] Sharma A, Pareek V, Zhang D. Biomass pyrolysis—A review of modelling, process parameters and catalytic studies. Renew Sustain Energy Rev. 2015; 50: 1081–1096.

[105] Imaizumi K. Forensic investigation of burnt human remains. Res Rep Forensic Med Sci. 2015; 5: 67–74.

[106] Dehaan JD. Fire and bodies In: Schmidt CW, Symes SA (Eds.), The analysis of burned human remains. London: Academic Press 2008; pp. 1–13.

[107] Eckert WG, James S, Katchis S. Investigation of cremation and severely burned bodies. Am J For Med Pathol. 1998; 9: 188–200. 10.1097/00000433-198809000-00002 3177344

[108] Holden JL, Phakey PP, Clement JG. Scanning electron microscope observations of incinerated human femoral bone: a case study. For Sci Int. 1995; 74:17–28. 10.1016/0379-0738(95)01734-z 7665129

[109] Ubelaker DH. The forensic evaluation of burned skeletal remains: A synthesis. Forensic Sci Int. 183: 1–5. 10.1016/j.forsciint.2008.09.019 19010619

[110] Pope EJ, Smith OC. Identification of traumatic injury in burned cranial bone: an experimental approach. J Forensic Sci. 2004; 49: 431–440. 15171155

[111] Black M, Graham DI. Sudden unexplained death in adults caused by intracranial pathology. J Clin Pathol. 2002; 55: 44–50. 11825924 10.1136/jcp.55.1.44 PMC1769576

[112] Kawasumi Y, Usui A, Hosokai Y, Sato M, Funayama M. Heat haematoma: post-mortem computed tomography. Clin Radiol. 2013; 68: e95–e97. 10.1016/j.crad.2012.10.019 23219455

[113] Goyal MK, Kochar SR, Asawa SS. Heat induced morphological changes in the brain. J Indian Acad For Med. 2010; 32: 75–77.

