# Supplementary figures and images for: A hypothesis of sudden body fluid vaporization in the 79 AD victims of Vesuvius
Source: PLoS One. 2018 Sep 26;13(9):e0203210. doi: 10.1371/journal.pone.0203210 (PMC6157861; doi:10.1371/journal.pone.0203210)

**
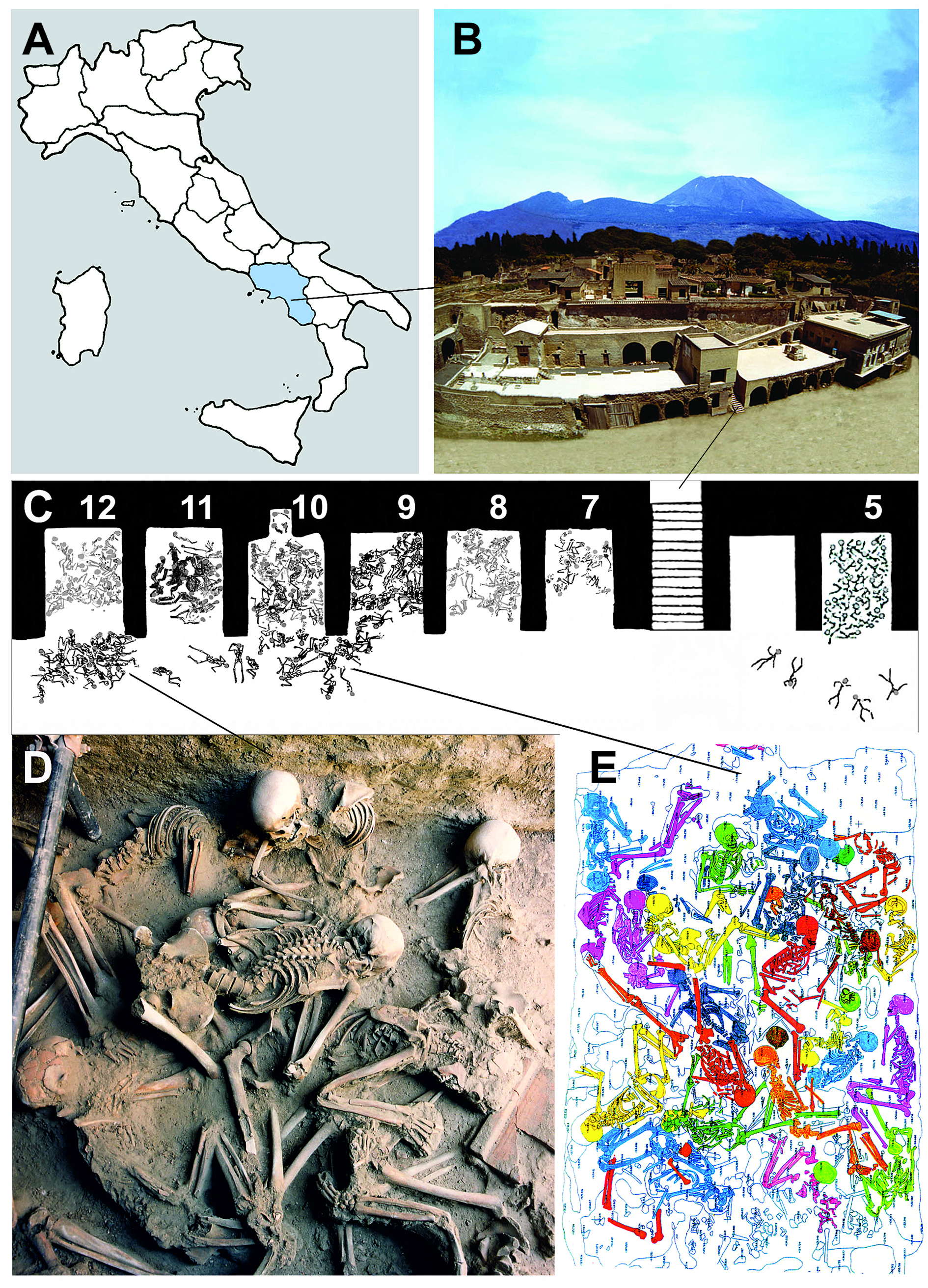
**

Supplement: S1 Fig — A. Map showing approximate location of the site; B. View of the archaeological excavations; C. The seafront chambers crowded with different numbers of victims’ skeletons; D. A group of victims unearthed in chamber 12; E. Planimetry of chamber 10 (The authors created the image themselves. Photos [B, D] and images [C, E] by P. Petrone). (DOCX) [file pone.0203210.s001.docx]

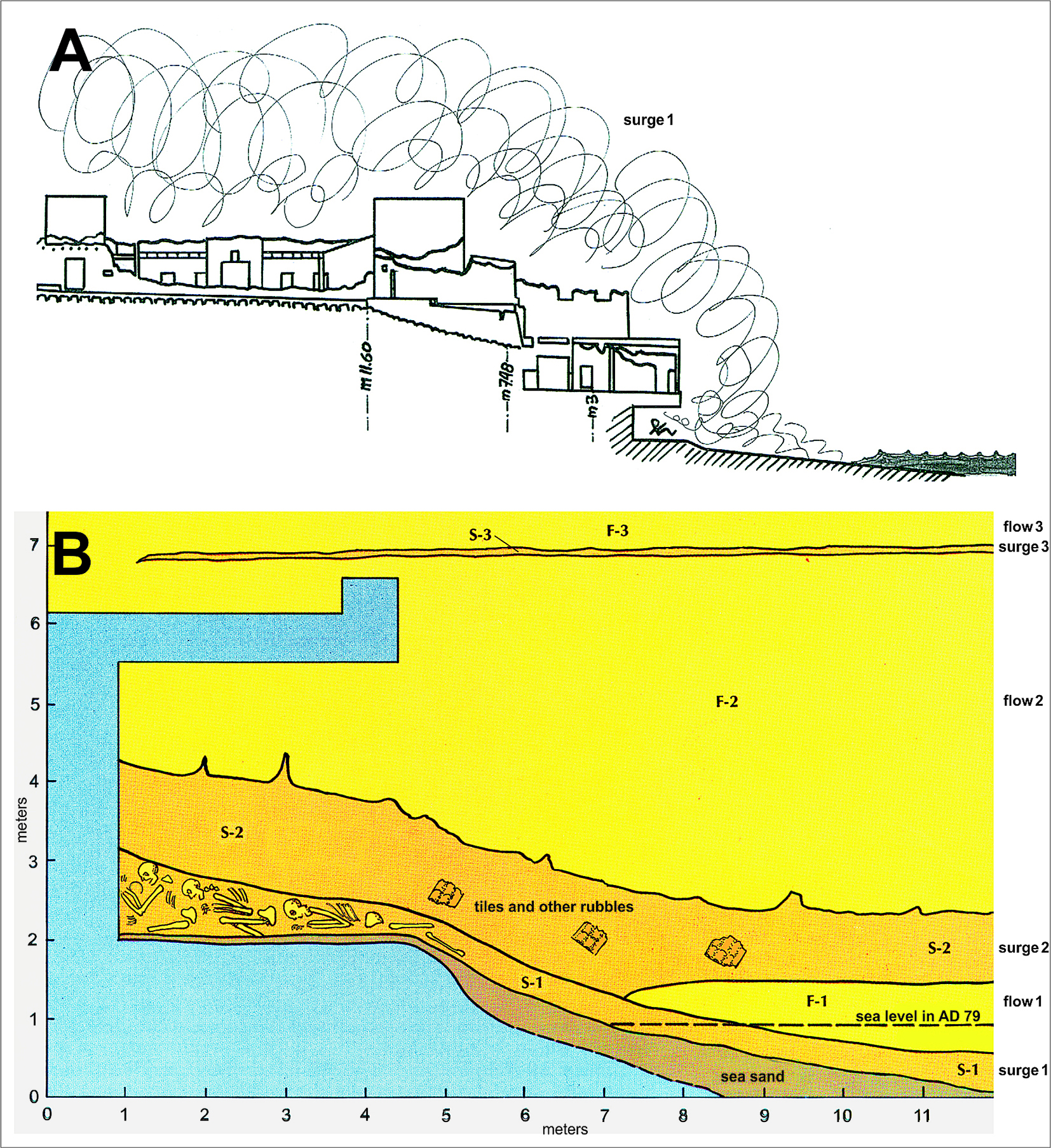

Supplement: S2 Fig — Planimetric sections of the town (A) and a boat-chamber (B). A. Section of the inferior IV cardo, the suburban area and the chambers facing the sea. Note the turbulent surge cloud passing through the town and emplacing on the beach and within the chambers; B. Section of the seafront area with the victims buried within the ash surge deposit (images modified from Budetta, 1993 and Sigurdsson et al., 1985). (DOCX) [file pone.0203210.s002.docx]

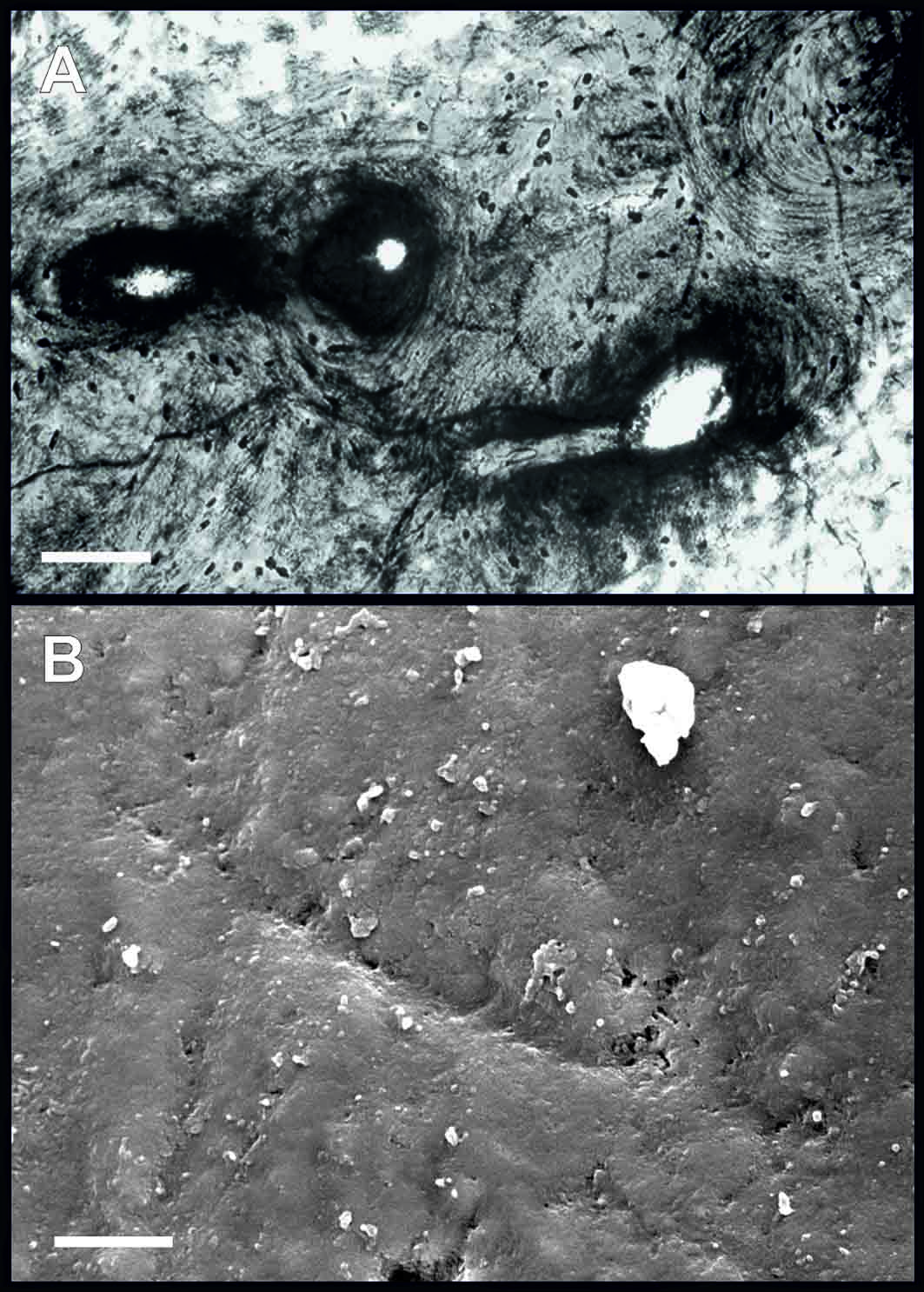

Supplement: S3 Fig — Adult bone analyzed with a light microscope (scale bar 100 μm) and a scanning electron microscope (scale bar 10 μm, 1700×): Radius showing both linear and polygonal cracking (A) and incipient recrystallization (B). At 500–800°C the basic bone structure recrystallizes into irregular globules (17). (DOCX) [file pone.0203210.s003.docx]

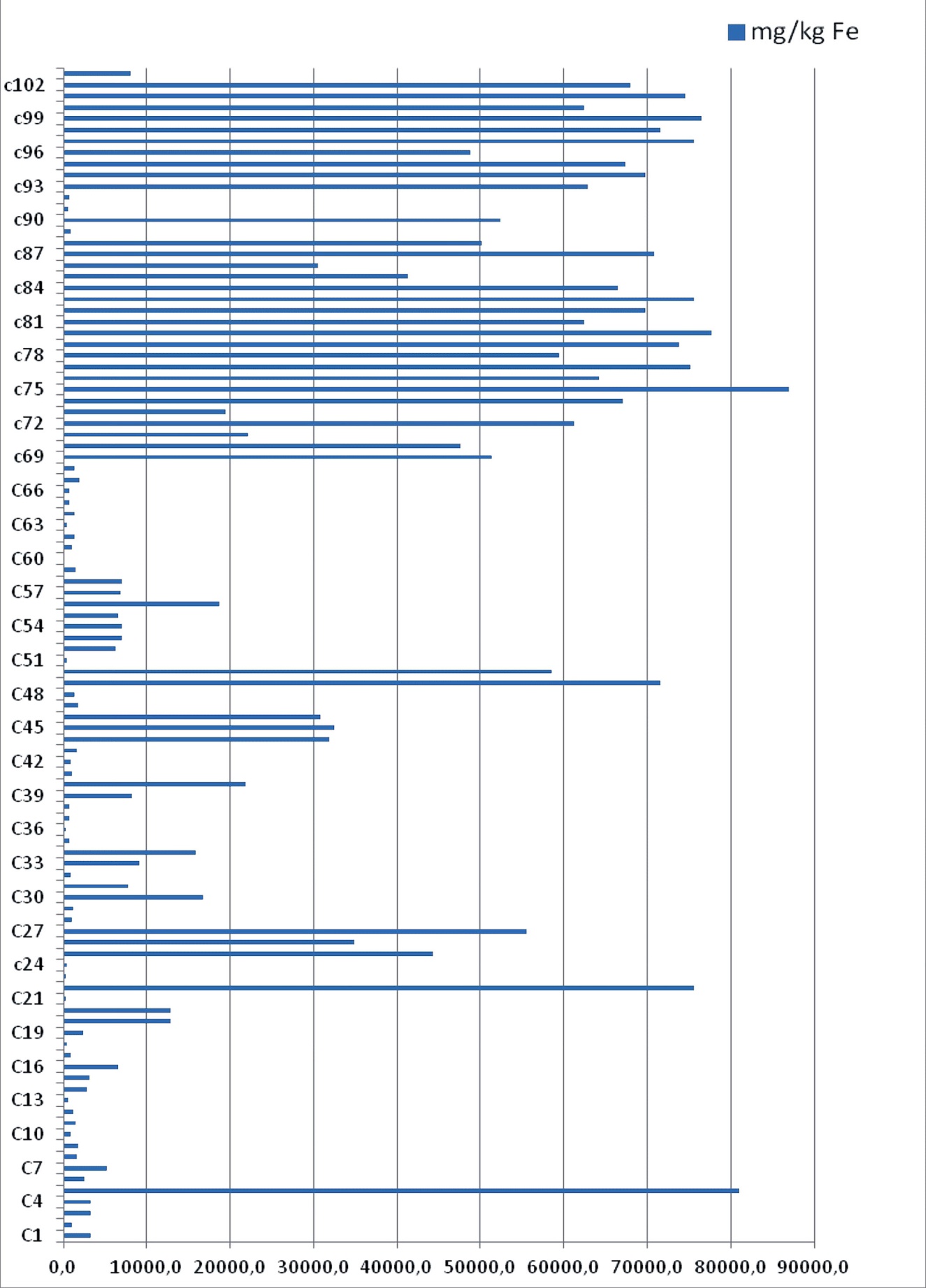

Supplement: S4 Fig — (DOCX) [file pone.0203210.s004.docx]

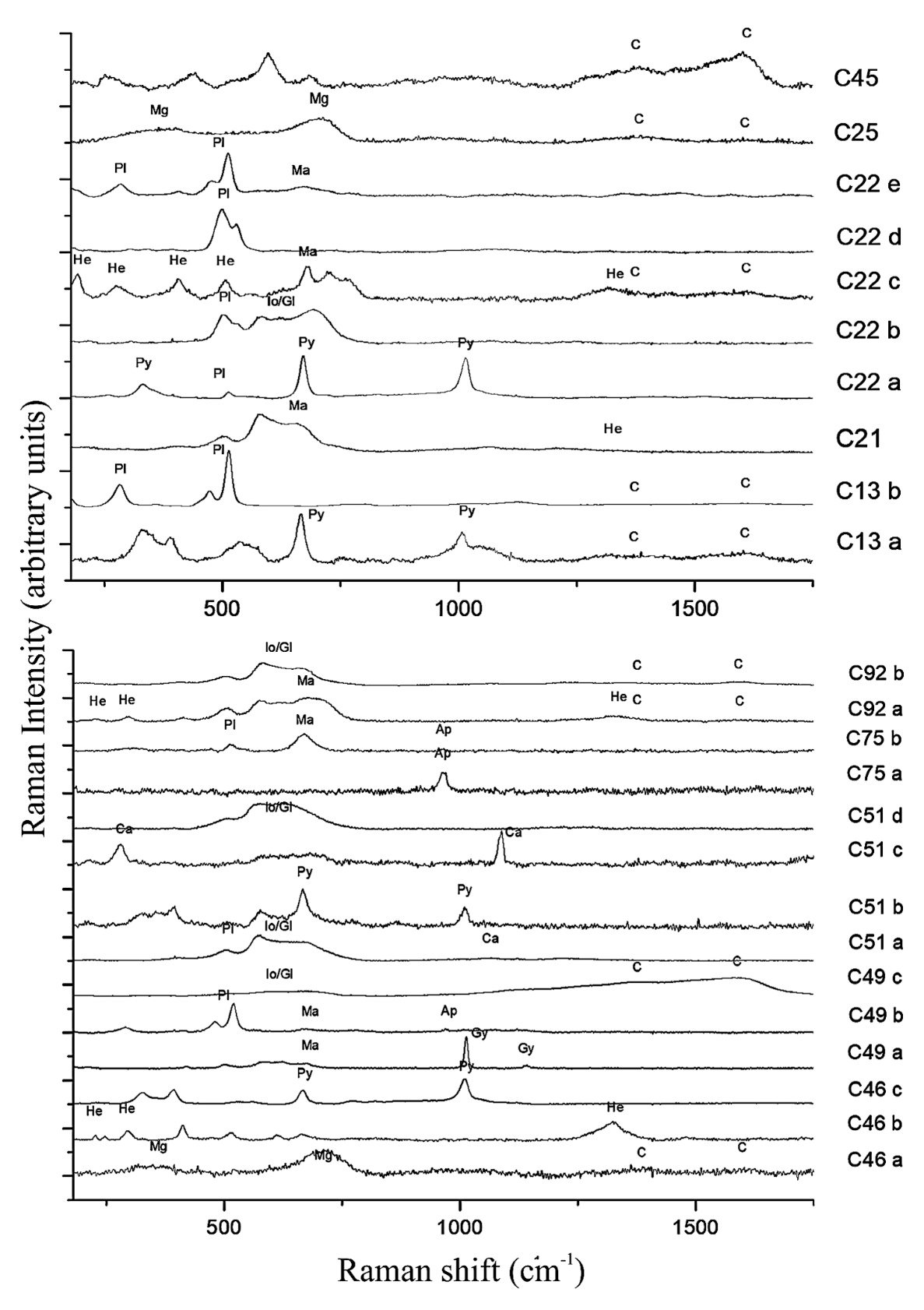

Supplement: S5 Fig — A. Samples 13, 21, 22, 25 and 45. Ap (apatite), C (amorphous carbon), Ca (Carbonate), He (hematite), Gl (SiO2 glasses), Io (mixture of multiple iron oxides), Ih (iron oxyhydroxides), Ma (Magnetite), Mg (maghemite), Pl (Plagioclase), Py (Pyroxene); B. samples 46, 49, 51, 75, 92. Ap (apatite), C (amorphous carbon), Ca (Carbonate), Gl (SiO2 glasses), Gy (gypsum), He (hematite), Io (mixture of multiple iron oxides), Ma (Magnetite), Mg (maghemite), Pl (Plagioclase), Py (Pyroxene). Frequencies have been reported in Table 2. (DOCX) [file pone.0203210.s005.docx]
