# Supplementary material for: A hypothesis of sudden body fluid vaporization in the 79 AD victims of Vesuvius
Source: PLoS One. 2018 Sep 26;13(9):e0203210. doi: 10.1371/journal.pone.0203210 (PMC6157861; doi:10.1371/journal.pone.0203210)
Supplement: S1 Table — Individual A was one of the very few victims found in the town. (DOCX) [file pone.0203210.s006.docx]

| **≠** | **chamber** | **ind** | **sex** | **age at death** | **≠** | **chamber** | **ind** | **sex** | **age at death** | **≠** | **chamber** | **ind** | **sex** | **age at death** |
| --- | --- | --- | --- | --- | --- | --- | --- | --- | --- | --- | --- | --- | --- | --- |
| **1** | EF5 | 1 | M | 14,0 | **28** | EF10 | 24 | F | 41,1 | **55** | EF12 | 8 | M? | 37,8 |
| **2** | EF5 | 2 | F | 20,2 | **29** | EF10 | 25 | M? | 21,5 | **56** | EF12 | 9 | F | 25,0 |
| **3** | EF5 | 3 | M | 20,2 | **30** | EF10 | 25b | ud | 21,5 | **57** | EF12 | 10 | M? | 9,0 |
| **4** | EF10 | 1 | M | 41,0 | **31** | EF10 | 26 | M? | 9,2 | **58** | EF12 | 11 | M | 52,3 |
| **5** | EF10 | 2 | M | 15,0 | **32** | EF10 | 27 | F? | 5,3 | **59** | EF12 | 12 | M? | 3,0 |
| **6** | EF10 | 3 | M | 48,0 | **33** | EF10 | 28 | F | 36,5 | **60** | EF12 | 13 | F? | 37,3 |
| **7** | EF10 | 4 | F | 29,1 | **34** | EF10 | 29 | F | 27,0 | **61** | EF12 | 14 | M | 12,0 |
| **8** | EF10 | 5 | M | 21,4 | **35** | EF10 | 30 | M? | 2,5 | **62** | EF12 | 15 | F | 32,7 |
| **9** | EF10 | 6 | M | 30,8 | **36** | EF10 | 31 | ud | 29,5 | **63** | EF12 | 16 | M | 37,2 |
| **10** | EF10 | 7 | M | 35,5 | **37** | EF10 | 32 | M? | 9,5 | **64** | EF12 | 17 | # | 5,5 |
| **11** | EF10 | 8 | M | 13,2 | **38** | EF10 | 33 | F | 12,0 | **65** | EF12 | 18 | F? | 3,5 |
| **12** | EF10 | 9 | M | 14,7 | **39** | EF10 | 34 | F? | 19,5 | **66** | EF12 | 19 | M | 33,9 |
| **13** | EF10 | 10 | M | 35,1 | **40** | EF10 | 35 | M | >20 | **67** | EF12 | 20 | M | 17,0 |
| **14** | EF10 | 11A | F | 31,3 | **41** | EF10 | 36 | M | 16,0 | **68** | EF12 | 21 | F | 32,1 |
| **15** | EF10 | 11B | M | 34,3 | **42** | EF10 | 37 | ud | 29,5 | **69** | EF12 | 22 | M | 20,5 |
| **16** | EF10 | 12 | M | 34,0 | **43** | EF10 | 38 | ud | 13,5 | **70** | EF12 | 23 | M | 42,0 |
| **17** | EF10 | 13 | M | 33,9 | **44** | EF10 | 39 | M | 13,5 | **71** | EF12 | 24 | M? | 9,5 |
| **18** | EF10 | 14 | M | 37,0 | **45** | EF10 | 40 | ud | 12,5 | **72** | EF12 | 25 | M | 10,5 |
| **19** | EF10 | 15 | F | 28,6 | **46** | EF10 | 41 | F? | 1,0 | **73** | EF12 | 26 | M | 31,0 |
| **20** | EF10 | 16 | F | 35,7 | **47** | EF11 | 15 | ud | 7 ium | **74** | EF12 | 27 | M | 40,0 |
| **21** | EF10 | 17 | M | 35,5 | **48** | EF12 | 1 | M? | 4,0 | **75** | EF12 | 28 | F | 30,4 |
| **22** | EF10 | 18 | F | 37,6 | **49** | EF12 | 2 | F | 28,0 | **76** | EF12 | 29 | F? | 12,0 |
| **23** | EF10 | 19 | M | 30,0 | **50** | EF12 | 3 | F | 26,8 | **77** | EF12 | 30 | F | 35,2 |
| **24** | EF10 | 20 | M | 44,0 | **51** | EF12 | 4 | M | 28,0 | **78** | EF12 | 31 | F? | >20 |
| **25** | EF10 | 21 | M | 38,0 | **52** | EF12 | 5 | M? | 16,6 | **79** | EF12 | 32 | ud | 3 |
| **26** | EF10 | 22 | M | 20,5 | **53** | EF12 | 6 | F? | 8,5 | **80** | town | A | F? | 5,5 |
| **27** | EF10 | 23 | M | 36,5 | **54** | EF12 | 7 | M? | 17,3 |  |  |  |  |  |
