# Supplementary material for: A hypothesis of sudden body fluid vaporization in the 79 AD victims of Vesuvius
Source: PLoS One. 2018 Sep 26;13(9):e0203210. doi: 10.1371/journal.pone.0203210 (PMC6157861; doi:10.1371/journal.pone.0203210)
Supplement: S2 Table — (DOCX) [file pone.0203210.s007.docx]

| **Sample N** | **Chamber** | **Specimen N** | **Type of sample** | **Source** |
| --- | --- | --- | --- | --- |
| **C 1** | 6 | / | Ash S1 | inside balsamar (Inventory N. 3441) |
| **C 2** | 6 | / | Ash S1 | inside balsamar (Inventory N. 3442) |
| **C 3** | 6 | / | Ash S1 | inside balsamar (Inventory N. 76797) |
| **C 4** | 10 | 26 | Ash S1 | endocranial ash cast |
| **C 5** | 10 | 26 | Red incrustations | endocranial ash cast |
| **C 6** | 10 | 26 | Dark-grey incrustations | endocranial ash cast |
| **C 7** | 10 | 14-49 | Ash S1 + Red incrustations | right shoulder and leg individuals 14-49 |
| **C 8** | 10 | / | Ash S1 | excavations 1997-1998 |
| **C 9** | 10 | 10 | Ash S1 | under left foot (level skeleton/ash) |
| **C 10** | 10 | 10 | Sand | under left foot (level ash/sand) |
| **C 11** | 10 | 10 | Ash S1 | under pelvis (level skeleton/ash) |
| **C 12** | 10 | / | Ash S1 | contact level ash/sand |
| **C 13** | 10 | / | Sand | contact level ash/sand |
| **C 14** | 10 | 39 | Ash S1 | under left foot (level skeleton/ash) |
| **C 15** | 10 | 24 | Bone + Ash | endocranial ash cast (S1 + S2?) |
| **C 16** | 10 | 2 | Ash S1 | endocranial ash cast |
| **C 17** | 12 | 27 | Carbonized residues | left radius and ulna |
| **C 18** | 10 | 27 | Carbonized residues | left scapula (rear wing) |
| **C 19** | 10 | 22 | Blackened Ash | left knee (level skeleton/ash) |
| **C 20** | 10 | 19 | Ash S1 + Red residues | left hand (level skeleton/ash) |
| **C 21** | 12 | 22 | Black incrustations | endocranial cavity |
| **C 22** | 12 | 5 | Sand + Red incrustations | level ash/sand |
| **C 23** | 10 | 17 | Black incrustations | thoracic vertebrae V, VI |
| **C 24** | 10 | 17 | Ash S1 + Black incrustations | under the skull |
| **C 25** | 10 | 19 | Red incrustations | left femur, right + left acetabulum |
| **C 26** | 10 | 5 | Ash S1 + Red incrustations | endocranial cavity |
| **C 27** | 11 | 15 | Red incrustations | coccyx (pelvis with 7m intra-uterine fetus) |
| **C 28** | 12 | 3 | Blackened sand | under right foot |
| **C 29** | 12 | 22 | Ash S1 + carbonized residues | from the corpse |
| **C 30** | 10 | 40 | Ash S1 + Red residues | under left foot (level skeleton/ash) |
| **C 31** | 12 | 14 | Sand + Red residues | left hand (level ash/sand) |
| **C 32** | 10 | 26 | Sand + carbonized residues | under the skull (level ash/sand) |
| **C 33** | 10 | Q2 b | Red residues | sporadic fragments |
| **C 34** | 10 | 39 | Ash S1 + Red residues | left under leg (level skeleton/ash) |
| **C 35** | 10 | 28 | Ash S1 + Red residues | left under leg (level skeleton/ash) |
| **C 36** | 12 | 22 | Black incrustations | endocranial cavity |
| **C 37** | 12 | 28 | Sand + Ash | iron object between right wrist/leg |
| **C 38** | 10 | 21 | Black incrustations | sporadic right hand/ankle |
| **C 39** | 10 | 4 | Ash S1 + Red incrustations | under lumbar vertebrae |
| **C 40** | 12 | ? | Ash S1 + iron metal residues | close to iron bracelet |
| **C 41** | 12 | / | Sand + black residues | level ash/sand |
| **C 42** | 12 | 27 | Sand + black residues | under right foot (level ash/sand) |
| **C 43** | 12 | 6 | Ash S1 | endocranial cavity |
| **C 44** | 10 | 3 | Ash S1 + Red incrustations | right ileum (level skeleton/ash) |
| **C 45** | 12 | 3 | Ash S1 + Red incrustations | under right foot (level ash/sand) |
| **C 46** | 10 | Sporadic-2 | Sand + Red incrustations | under pelvis and legs |
| **C 47** | 10 | 24 | Ash S1 | endocranial ash cast |
| **C 48** | 12 | 24 | Ash S1 | endocranial ash cast (frontal area) |
| **C 49** | 12 | 24 | Red incrustations | endocranial ash cast (frontal bone) |
| **C 50** | 11 | 15bis | Red incrustations | supraorbital area (7m intra-uterine fetus) |
| **C 51** | 10 | 11A | Black incrustations | partially carbonized sacrum |
| **C 52** | 12 | 9 | Ash S1 | endocranial cavity |
| **C 53** | 10 | 14/19 | Ash S1 | between bones of individuals 14 and 19 |
| **C 54** | 5 | 3 | Ash S1 | superior maxilla |
| **C 55** | 6 | / | Ash S1 | inside balsam (Inventory N. 78738) |
| **C 56** | 5 | 3 | Red incrustations | teeth (left hemi maxilla) |
| **C 57** | 5 | 3 | Black incrustations | left hand (phalanxes + metacarpals) |
| **C 58** | 12 | 11 | Ash S1 | nasal cavity |
| **C 59** | Sac-Aug | guardian | Black incrustations | thorax, vertebral column |
| **C 60** | Sac-Aug | guardian | Black incrustations | thorax, vertebral column |
| **C 61** | Sac-Aug | guardian | Black incrustations | right femur |
| **C 62** | Sac-Aug | guardian | Black incrustations | medial side right leg |
| **C 63** | Sac-Aug | guardian | Black incrustations | right pelvis |
| **C 64** | Sac-Aug | guardian | Black incrustations | abdomen |
| **C 65** | Sac-Aug | guardian | Black incrustations | right hemi mandible |
| **C 66** | Sac-Aug | guardian | Black incrustations | right hemi mandible |
| **C 67** | Sac-Aug | guardian | Ash | between legs, close to sacrum |
| **C 68** | Sac-Aug | guardian | Black incrustations | close to wooden bed |
| **C 69** | 5 | 1 | Red incrustations | femur (periosteum + proximal epiphysis) |
| **C 70** | 5 | 3 | Red incrustations | branch of the jaw |
| **C 71** | 10 | Sporadic-1 | Black + Red incrustations | sporadic skull (between individuals 3, 5, 7) |
| **C 72** | 10 | Sp-3 Q2 a-b | Red incrustations | humerus (endosteal surface) |
| **C 73** | 10 | Sp-4 Q2 b | Dark Red incrustations | sporadic rib |
| **C 74** | 10 | Sp-5 Q2 b-9 | Red incrustations | sporadic rib |
| **C 75** | 10 | Sp-6 Q2 b-1c | Red incrustations | skull (sporadic, external + internal surface) |
| **C 76** | 10 | Sp-7 Q2 b-1c | Red incrustations | sporadic fragmentary bones |
| **C 77** | 10 | Sp-8 Q2 b-1c | Red incrustations | sporadic vertebrae |
| **C 78** | 10 | Sp-9 Q2 b-1c | Red incrustations | fragments long bones (endosteal surface) |
| **C 79** | 10 | Sp-10 Q2 b-1c | Red incrustations | long bone epiphysis (trabecular bone) |
| **C 80** | 10 | Sp-11 Q3a-1VLI | Red incrustations | fragmentary sporadic bones |
| **C 81** | 10 | Sp-12 Q3 a | Red incrustations | sporadic scapula (glenoid cavity) |
| **C 82** | 10 | Sp-13 Q3 a | Red incrustations | fragmentary sporadic bones |
| **C 83** | 10 | 1 | Red incrustations | skull cap (endosteal surface) |
| **C 84** | 10 | 1 | Red incrustations | rib (periosteum) |
| **C 85** | 10 | 2 | Red incrustations | sacrum |
| **C 86** | 10 | 3 | Red incrustations | rib (periosteum) |
| **C 87** | 10 | 3 | Red incrustations | 3 vertebrae |
| **C 88** | 10 | 4 | Red incrustations | clavicle (periosteum) |
| **C 89** | 10 | 10 | Red material | cloth tissue? |
| **C 90** | 10 | 12 | Red incrustations | radium (periosteum) |
| **C 91** | 10 | 16 | Black incrustations | radium (periosteum) |
| **C 92** | 10 | 17 | Black + Red incrustations | scapula + clavicle (sternal epiphysis) |
| **C 93** | 10 | 18 | Red incrustations | heel |
| **C 94** | 10 | 18 | Red incrustations | rib (endosteum, trabecular bone) |
| **C 95** | 10 | 19 | Red incrustations | rib, manubrium, sternum, clavicle |
| **C 96** | 10 | 20 | Red incrustations | femur (endosteum, trabecular bone) |
| **C 97** | 10 | 38 | Black + Red incrustations | metatarsals (right foot) |
| **C 98** | 12 | 13 | Red incrustations | rib (endosteum, trabecular bone) |
| **C 99** | 12 | 16 | Red incrustations | rib + sternum (periosteum) |
| **C 100** | 12 | 17 | Dark Red incrustations | rib (periosteum) |
| **C 101** | 12 | 20 | Red incrustations | 3 ribs (periosteum) |
| **C 102** | 12 | 28 | Red incrustations | rib (endosteum, trabecular bone) |
| **C 103** | 12 | 30 | Ash S1 + Red incrustations | under right ileum |
