# Supplementary material for: A hypothesis of sudden body fluid vaporization in the 79 AD victims of Vesuvius
Source: PLoS One. 2018 Sep 26;13(9):e0203210. doi: 10.1371/journal.pone.0203210 (PMC6157861; doi:10.1371/journal.pone.0203210)
Supplement: S3 Table — (DOCX) [file pone.0203210.s008.docx]

| **Sample pre-treatment** | **Protein Name (UniProtKB ID)** | **Peptides** |
| --- | --- | --- |
| **Urea 6M** | Collagen alpha-2(I) (P08123) | GIPGPVGAAGATGAR + Hydroxylation (P)GAPGAVGAPGPAGATGDRGEAGAAGPAGPAGPR + 2 Hydroxylation (P) |
|  | Collagen alpha-1(I) (P02452) | GSAGPPGATGFPGAAGR + 2 Hydroxylation (P) |
| **TFA-Acetonitrile** | Collagen alpha-2(I) (P08123) | GAPGAVGAPGPAGATGDRGEAGAAGPAGPAGPR + 2 Hydroxylation (P) |
|  | Collagen alpha-1(I) (P02452) | GVQGPPGPAGPR + Hydroxylation (P)GSAGPPGATGFPGAAGR + 2 Hydroxylation (P) |
| **RIPA** | Collagen alpha-1(I) (P02452) | GSAGPPGATGFPGAAGR + 2 Hydroxylation (P)GLTGPIGPPGPAGAPGDKGESGPSGPAGPTGAR + Hydroxylation (P), (K) |
| **CH_3_Cl_3_/CH_3_OH** | Collagen alpha-2(I) (P08123) | GIPGPVGAAGATGAR + Hydroxylation (P)GAPGAVGAPGPAGATGDRGEAGAAGPAGPAGPR + 2 Hydroxylation (P) |
|  | Collagen alpha-1(I) (P02452) | GSAGPPGATGFPGAAGR.V + 2 Hydroxylation (P) |
